# IMPIPS: The **Im**mune **P**rotection-**I**nducing **P**rotein **S**tructure Concept in the Search for Steric-Electron and Topochemical Principles for Complete Fully-Protective Chemically Synthesised Vaccine Development

**DOI:** 10.1371/journal.pone.0123249

**Published:** 2015-04-16

**Authors:** Manuel Elkin Patarroyo, Adriana Bermúdez, Martha Patricia Alba, Magnolia Vanegas, Armando Moreno-Vranich, Luis Antonio Poloche, Manuel Alfonso Patarroyo

**Affiliations:** 1 Fundación Instituto de Inmunología de Colombia (FIDIC), Bogotá, Colombia; 2 Universidad Nacional de Colombia, Bogotá, Colombia; 3 Universidad del Rosario, Bogotá, Colombia; National Chiao Tung University, TAIWAN

## Abstract

Determining immune protection-inducing protein structures (IMPIPS) involves defining the stereo-electron and topochemical characteristics which are essential in MHC-p-TCR complex formation. Modified high activity binding peptides (mHABP) were thus synthesised to produce a large panel of IMPIPS measuring 26.5 ±3.5Å between the farthest atoms fitting into Pockets 1 to 9 of HLA-DRβ1* structures. They displayed a polyproline II-like (PPII_L_) structure with their backbone O and N atoms orientated to establish H-bonds with specific residues from HLA-DRβ1*-peptide binding regions (PBR). Residues having specific charge and *gauche^+^* orientation regarding p3χ1, p5χ2, and p7χ1 angles determined appropriate rotamer orientation for perfectly fitting into the TCR to induce an appropriate immune response. Immunological assays in *Aotus* monkeys involving IMPIPS mixtures led to promising results; taken together with the aforementioned physicochemical principles, non-interfering, long-lasting, protection-inducing, multi-epitope, multistage, minimal subunit-based chemically-synthesised peptides can be designed against diseases scourging humankind.

## Introduction

Chemical and physical knowledge accumulated over the last five decades at subatomic level regarding the microbe’s most relevant molecules involved in invasion and infection and the human host’s immune system molecules’ biological and structural information has provided a solid background for promoting chemically-synthesised vaccines. Chemistry must thus be intrinsically involved in the task of developing new vaccines for humankind, adopting and adapting previously described principles or rules of chemistry as the basis for presenting the **i**mmune **p**rotection-**i**nducing **p**rotein **s**tructure (IMPIPS) concept.

Our group chose *Plasmodium falciparum* malaria as the prototype disease in the search for a logical and rational methodology for vaccine development since malaria is an acute disease that can be easily diagnosed, cured with pharmacological products and has an appropriate experimental model (the *Aotus* monkey). Malaria produces ~200 million cases and ~1.0 million deaths per year [1], thereby making it one of humankind’s main public health problems.

The very frustrating results reported during the last four decades regarding biologically-derived, antimalarial vaccines [2–4] have thus forced in-depth analysis of some other alternatives. Such disappointments have included efforts to use recombinant proteins [5], naked DNA fragments [6], vector-based [7], whole attenuated, genetically-modified parasites [8] for immunising thousands of people. This has consistently led to negative results or heroic approaches involving intravenously immunising five times with 135,000 highly purified, live non-replicating (X-ray irradiated) sporozoites (Spz) to provide some protection [9].

The original, chemically-synthesised, anti-*P*. *falciparum* vaccine (SPf66) was developed by our institute 27 years ago [10,11]; it consisted of a polymerised chimeric polypeptide having aminoacid sequences derived from four different proteins [12–16]. Its protective immunity lasted for several years [17] and highlighted the feasibility and advantages of chemically-synthesised vaccines. The complexity of the *P*. *falciparum* parasite, due to the large set of proteins involved and the mechanisms involved in the invasion of host cells and immune evasion, also had to be taken into account.

Precise data gathered during the last 25 years based on ~40,000 synthesised malarial peptides (~4,000 of them having been tested in the *Aotus* monkey model) has led to a thoroughly described decalogue of chemical and physical principles for individually sterile, protective immunity-inducing synthetic peptides [18–20]. They were selected using a functional-structural approach stating that many (multi-epitope) specifically modified chemically-synthesised peptides, should be used as components of a fully-protective definitive vaccine against this deadly disease; they have been called modified high activity binding peptides (mHABPs), derived from conserved high activity binding peptides (cHABPs) which are functionally-relevant in invasion during the parasite’s developmental stages (i.e. multistage) [19,20]. The physical-chemical principles described have thus been used for ^1^H-NMR analysis of a large set of *P*. *falciparum* mHABPs at the subatomic level. ^1^H-NMR has also been used for thoroughly demonstrating a ~26.5 Å ±3.5 Å inter-atom distance from position 1 to 9 to fit into MHCII Pockets 1 to 9 in highly immunogenic protection-inducing (HIPI) and very high, long-lasting antibody-inducing (VHLLAI) mHABPs, according to already identified HLA-DRβ1* binding capacity, binding motifs and binding registers, this being a fundamental principle for IMPIPS development [19–21].

Logical and rational vaccine development must include all the 3D structural information regarding a parasite’s molecules involved in host cell invasion to render them highly immunogenic and protection-inducing (mHABPs). The 3D structure of the major histocompability complex class II (MHCII) molecules must also be ascertained to enable mHABPs to fit perfectly into it and mHABP presentation to the T-cell receptor (TCR) (known as the MHC II-p-TCR) [22–27].

TCR α- and β-chains contain complementary determining regions (CDR) to scan first and then bind to the MHCII-peptide complex to form the appropriate tri-molecular MHCII-peptide-TCR complex (MHCII-p-TCR) and properly activate the immune system [28–31]. Physicochemical rules regarding such tri-molecular complex formation had thus to be defined regarding protective immunity. Our group has recently used ^1^H-NMR to demonstrate that most Spz-derived VHLLAI 3D structures and those derived from merozoites (Mrz) (i.e. HIPI) displayed or contained polyproline II-like left-handed (PPII_L_) helixes to establish H-bonds or van der Waals (vdW) interactions with specific residues in MHC II [32,33], as shown in antigenic peptide structures by X-ray crystallography [34]. mHABPs having *gauche* ^*+*^ orientation in positions 3 and 7 (henceforth position is represented by p, i.e. p3 and p7) of PBR fitting residues led to establishing the atomic basis for long sought-after vaccine development principles [21] (i.e. long-lasting, antibody/protection-inducing).

The highly relevant circumsporozoite protein (CSP), and thrombospondin-related protein (TRAP) mHABPs were thus chosen from among ~20 Spz-derived molecules involved in hepatocyte invasion as the **first line of defence** [35]. Apical Mrz antigen 1 (AMA-1), Mrz surface protein 2 (MSP-2), erythrocyte binding antigen 175 (EBA-175) and serine repeat antigen 5 (SERA-5) mHABPs were selected as components of the **second line of defence** from among the ~50 Mrz proteins involved in invasion of RBC [18–20]. Their cHABP, mHABP (shown in bold hereafter) amino acid sequences, as well as their immunogenicity, as assessed by the immunofluorescence antibody, IFA or Western blot tests, and protection-inducing ability, have been individually and convincingly demonstrated in many monkey trials and studies [18–20,35], serving as the basis for the IMPIPS concept and methodology.

Attention regarding the Spz stage has been focused on VHLLAI anti-Spz-derived mHABPs as assessed by ELISA (data not shown), immunofluorescent antibody (IFA) test and Western blot (WB) analysis due to unpredictable results regarding the protection of *Aotus* monkeys during Spz challenge via a naturally-infected *Anopheles* mosquito bite. CSP **32958** (4388) and **25608** (4383) and TRAP **24246** (3287) and **24254** (3347) were highly immunogenic for *Aotus* monkeys determined by each inducing high antibody titres (IFA titres ≥ 1:160 dilution) in several monkey trials [21] (8–10 monkeys each trial).

*Aotus* monkey trials are of seminal importance in developing synthetic vaccines since they completely simulate mHABP performance in the human population because the adopted animal model (*Aotus* monkeys) has a 90%-100% similar immune system molecules to that of humans (sometimes identical), as determined at DNA level [36–40]. The HLA-DRβ1*-like or *Aona*-DR genetic regions of ~400 *Aotus* have been DNA sequenced by our Institute, providing strong support for the above results [36–40] and mHABP potential as vaccine component for immediate human use. Sets of peptide mixes were thus inoculated into groups of *Aotus* monkeys to evaluate the efficacy of the aforementioned IMPIPS-based approach.

## Results and Discussion

### VHLLAI mHABP in complete antimalarial vaccine mixture development

Since complete protective immunity is a very complex mechanism involving many mHABPs (multi-antigenic) derived from numerous proteins involved in host invasion during several development stages (multistage), we started the search for appropriate mHABP **mixtures**, trying to include most HLA-DRβ1* allele-specific ones. It is thus shown here that fully protective complete IMPIPS mHABP mixture composition for vaccine development follows electron-steric-topochemical rules.

We have recently reported >25 *Aotus* trials including previously-identified highly immunogenic CSP- and TRAP- and other Spz-derived mHABP mixtures which involved immunising 8 to 10 monkeys per trial [21] in the search for an appropriate anti-Spz mHABP **mixture** covering most Spz proteins and HLA-DRβ1*-like variants. Individual high antibody reactivity (determined by IFA and WB) disappeared in most monkey trials when some mixtures were used, suggesting competition [41], blocking [42] or suppression [43,44]; this represents an insurmountable immunological phenomena for which no logical explanation has been provided to date, thereby representing a great limitation for vaccine development.

The first suggestion for a way to overcome this problem appeared when TRAP-derived **24246**+ and CSP-derived **25608**+**32958** mHABPs were mixed (**Table 1**, group A-01/12) and very high IFA-assessed anti-Spz antibody titres were detected in 40% of the immunised monkeys that lasted for at least 140 days after the first immunisation (80 days after 3^rd^ or III80). Analysis of Spz mHABP 3D structure (previously determined by ^1^H-NMR) led to finding *gauche* ^*+*^ orientation for p3 in mHABPs in the HLA-DRβ1* PBR during our search for a structural-immunological association. This striking result contrasted with around 30 trials (involving ~300 monkeys, including controls) involving other individually highly immunogenic mHABP mixtures where one or several mHABPs had *gauche* ^*-*^ or *trans* orientation in p3 and p7 and no antibodies were produced, suggesting blocking, suppression or immunological competition induced by this residue’s inappropriate rotamer orientation in some mHABPs.

**Table 1:** Immunological studies.

**Group A-01/12: Spz (24246, 25608, 32958)**						
***Aotus***	**PI**	**II10**	**III20**	**III80**		
**2–004**	**0**	**ND**	**5120**	**2560**		
**2–013**	**0**	**ND**	**2560**	**2560**		
**2–023**	**0**	**ND**	**160**	**0**		
**2–035**	**0**	**ND**	**5120**	**160**		
**2–044**	**0**	**ND**	**160**	**0**		
**2–051**	**0**	**ND**	**0**	**0**		
**2–056**	**0**	**ND**	**2560**	**2560**		
**2–066**	**0**	**ND**	**640**	**640**		
**Group B-12: Spz (25608, 32958, 24254, 24246)**						
***Aotus***	**PI**	**II10**	**III10**	**III20**	**III60**	**III180**
**2–360**	**0**	**1280**	**1280**	**1280**	**1280**	**1280**
**2–394**	**0**	**320**	**320**	**320**	**320**	**2560**
**2–449**	**0**	**640**	**160**	**320**	**320**	**160**
**2–580**	**0**	**640**	**0**	**160**	**160**	**0**
**2–645**	**0**	**640**	**-**	**-**	**-**	**-**
**2–732**	**0**	**0**	**0**	**0**	**320**	**0**
**2–786**	**0**	**0**	**0**	**0**	**0**	**0**
**Group C-12: Spz (24246 + 25608 + 32958) + Mrz (10022)**						
**IFA Spz**				**IFA Mrz**		
***Aotus***	**PI**	**II10**	**III20**	**PI**	**II10**	**III20**
**2–009**	**0**	**ND**	**0**	**0**	**0**	**0**
**2–080**	**0**	**ND**	**0**	**0**	**0**	**0**
**0–113**	**0**	**ND**	**0**	**0**	**0**	**0**
**2–123**	**0**	**ND**	**5120**	**0**	**0**	**0**
**2–160**	**0**	**ND**	**0**	**0**	**0**	**0**
**2–178**	**0**	**ND**	**0**	**0**	**0**	**160**
**2–211**	**0**	**ND**	**2560**	**0**	**0**	**0**
**2–234**	**0**	**ND**	**2560**	**0**	**0**	**0**
**Group D-13:**						
**IFA Spz**			**IFA Mrz**			
***Aotus***	**PI**	**II20**	**PI**	**II10**	**II20**	**II60**
**3–291**	**0**	**0**	**0**	**0**	**0**	**0**
**3–297**	**0**	**0**	**0**	**0**	**0**	**0**
**3–303**	**0**	**640**	**0**	**0**	**0**	**0**
**3–311**	**0**	**0**	**0**	**160**	**320**	**320**
**3–321**	**0**	**0**	**0**	**0**	**160**	**160**
**3–329**	**0**	**0**	**0**	**0**	**0**	**0**
**3–336**	**0**	**0**	**0**	**0**	**0**	**0**
**3–345**	**0**	**0**	**0**	**0**	**0**	**0**
**3–348**	**0**	**0**	**0**	**640**	**320**	**320**
**3–353**	**0**	**0**	**0**	**0**	**0**	**40**
**3–357**	**0**	**0**	**0**	**0**	**0**	**40**
**3–362**	**0**	**320**	**0**	**160**	**160**	**160**
**3–368**	**0**	**320**	**0**	**640**	**640**	**640**
**3–372**	**0**	**0**	**0**	**0**	**0**	**0**
**3–382**	**0**	**640**	**0**	**0**	**0**	**0**
**3–387**	**0**	**640**	**0**	**0**	**0**	**160**
**3–394**	**0**	**0**	**0**	**0**	**0**	**0**
**3–396**	**0**	**320**	**0**	**0**	**0**	**0**

Anti-Spz and anti-Mrz antibody titres (IFA), induced by specific mHABP mixtures, as explained in the text.

Another trial involved 7 *Aotus* immunised with a CSP-derived **25608**+**32958**+ and TRAP-derived **24246**+**24254** mHABP mixture, having *gauche* ^+^ orientation in p3 (**Table 1**, group B/12); this mixture continued being highly immunogenic in this trial in 40% of the monkeys, inducing very high antibody titres which lasted for at least 240 days after the first immunisation (III180).

**Fig 1:**
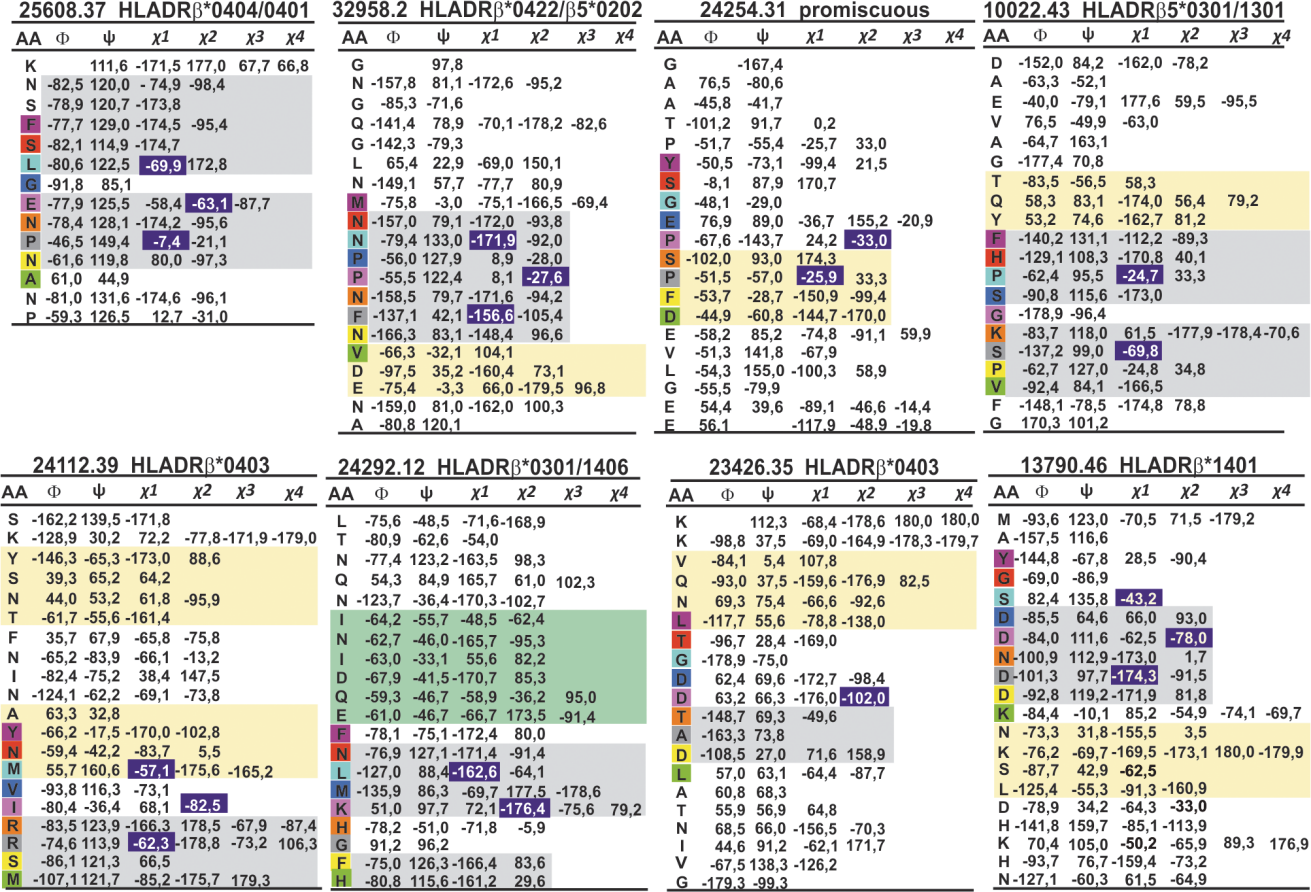
Representative torsion angles (Φ, Ψ, χ1, χ2, χ3 and χ4) in mHABPs involved in mixtures immunised in *Aotus* monkeys. Grey shows the PPII_L_ region, yellow shows the β-turn region and green an α-helix conformation. Purple box, χ1 angles for p3 and p7, and χ2 angles for p5, highlighting their *gauche+* rotamer orientation. The colours of residues vertically displayed in each mHABP correspond to the code for **Fig 3**.

An appropriate mixture of Spz-derived VHLLAI mHABPs having *gauche* ^*+*^ orientation in p3 and controlled by HLA-DRβ1* 0404/0401 or DRβ1*11 and 0403-like alleles covering 40% of the wild *Aotus* population (**Fig 1**) has thus enabled overcoming the competition, interference or suppression associated with vaccine mixture composition development.

### Recognising mHABP mixture-induced antibody reactivity with recombinant fragments (WB)

**Fig 2:**
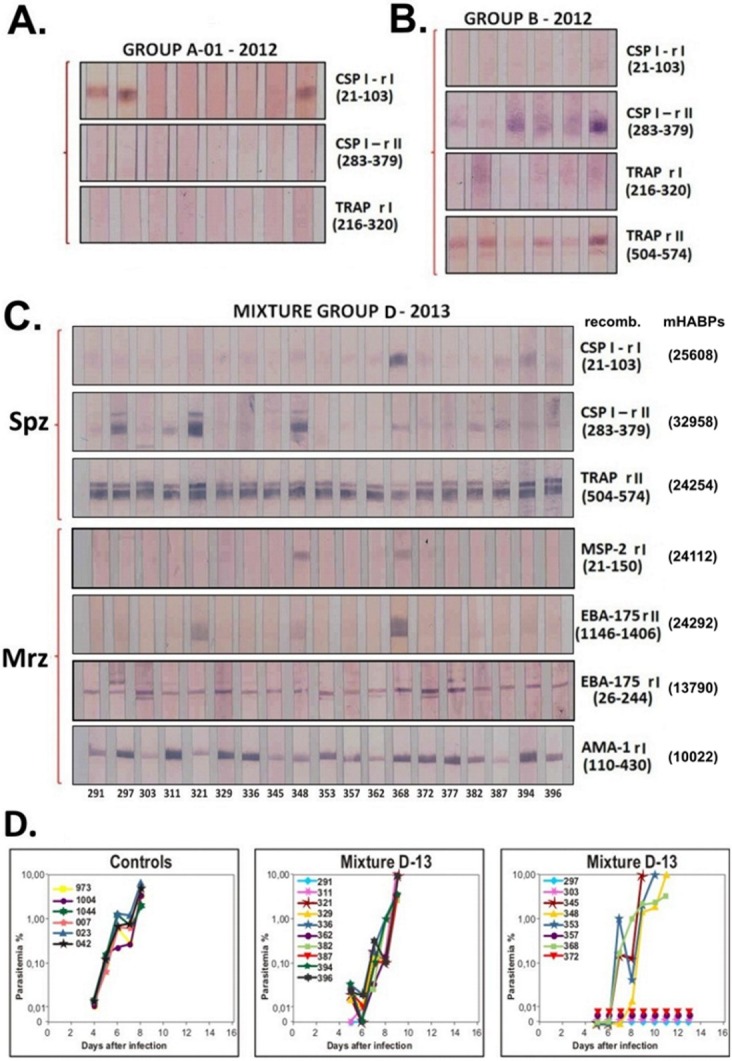
WB analysis showing the reactivity of *Aotus* immunised with different mixtures of mHABPs. **A.** Reactivity with CSP-derived **25608**, **32958**, and TRAP **24246** mHABP mixtures 140 days after first immunisation (80 days after 3^rd^ or III80) showing reactivity in 2/8 *Aotus* with CSP-rI. **B.** Reactivity with CSP-derived **25608**, **32958**, and TRAP **24254** and **24246** mHABP mixtures evidencing reactivity with CSP-rII and with TRAP rII in 5/6 *Aotus* monkeys **C.** Reactivity with CSP-derived rI **25608**, rII **32958**, TRAP rII **24254**, MSP-2 **24112,** EBA-175 RI **13790** and rII **24292** and AMA-1 **10022**.

All monkey sera were used at 1:25 dilution for WB analysis. Three out of the eight monkeys’ sera immunised with the **25608**+**32958**+**24246** mixture in monkey trial A-01/12 reacted very strongly with CSP1 rI but very weakly with CSP1 rII in one monkey, no reactivity being observed with the TRAP rI fragment (**Fig 2A**). The opposite occurred in trial B/12 involving the **25608** + **32958** + **24246** +**24254** mixture when weak reactivity occurred in 1/6 monkeys with CSP1-rI and strong reactivity with CSP rII in 3/6 monkeys (**Fig 2B**). Such variation may have been due to AoDRβ1* allele diversity in the small groups of monkeys in each trial; it should be noted that these were outbred trapped *Aotus* monkeys from different parts of the Colombian Amazon basin simulating HLA-DRβ1* genetic frequency distribution in the human population and that each immunological test detected different immunological characteristics (IFA native proteins and WB denatured molecules).

TRAP II reactivity was very strong in 5/6 monkeys in group B; however, no reactivity was observed in these monkeys with TRAP rI when **24246** was used and therefore this mHABP was not included in further mixtures (**Fig 2A** and **2B**).

Monkey trial D-13, including a larger number of monkeys (nineteen), was very informative since their sera reactivity with CSP1-rI was very strong in 1/19 monkeys and weak in two; CSP1-rII reactivity was very strong in 3/19 and weak in 4/19, while TRAP-rII reactivity was very strong in ALL 19 monkeys’ sera (**Fig 2C**). It is worth mentioning that ALL pre-immune sera were negative with Spz by IFA and WB when confronted with these recombinant fragments.

This data clearly suggested that while **25608** (4383) and **32958** (4388) were genetically controlled by AoDRβ1* alleles: HLA-DRβ1*1501/0401/0404 for **25608** and HLA-DRβ1*0422/β5*0202 like for **32958** [21,45], TRAP **24254** (3347) could have been a “universal epitope” [46–48] binding to either a large number of HLA-DRβ1*PBRs but mainly to HLA-DRβ1*0401 [49] or having different rotamer orientation in upwardly-pointing residues to contact the TCR thereby making it a “promiscuous epitope” [48].

Since Spz challenge in *Aotus* monkeys with the only *Aotu*s-adapted *P*. *falciparum* strain (Santa Lucia) able to infect these monkeys with *Anopheles* mosquito’s bite have displayed very weird and irreproducible results, the only surrogates (as a proof of concept) for these mHABPs’ immunogenicity is their induction of high antibody titres as assessed by IFA and WB reactivity of these monkeys’ sera with corresponding recombinant proteins (**Fig 2**).

### Anti-Mrz high immunogenicity and sterile protective immunity

Attention thus became focused on Mrz-derived mHABPs in the ongoing search for a complete tailor-made vaccine.

**H**igh **i**mmunogenicity associated with full-**p**rotective **i**mmunity (HIPI) has been induced in monkeys immunised with individual mHABPs (usually 5–8 per group) when challenged with the highly-virulent *Aotus*-adapted *P*. *falciparum* FVO strain via intravenous infection with 100,000 infected erythrocytes (Ei) (freshly obtained from another infected monkey) when the course of their infection (parasitaemia) was assessed by very sensitive acridine orange staining [10,19,20].

Disappointing results were obtained in more than 50 monkey trials (6–8 monkeys in each) which involved mixing Mrz-derived HIPI mHABPs, thereby confirming observations concerning Spz-derived mHABP mixtures. Based on previous findings concerning Spz-derived **24246**+**25608**+**32958** mHABPs being mixed with Mrz-derived **10022,** all having p3 and p7 *gauche* ^*+*^ orientation (**Table 1**, group C-2012), three out of the eight monkeys (~37%) developed very high antiSpz antibody titres (as previously observed with this Spz mixture), and one out of eight monkeys developed high IFA antiMrz antibody titres, as has always occurred when monkeys have been immunised with this mHABP (**10022**).

These results clearly suggested that Mrz-derived peptides having such p3 and p7 rotamer characteristics did not block, interfere, compete or suppress the immune response induced by the Spz mixture, maintaining their anti-Mrz antibody induction reactivity, confirmed later on by their corresponding Mrz recombinant fragments.

A cautious approach was thus adopted concerning the previously-described Spz-derived mixture which involved mixing more Mrz-derived mHABPs having *gauche* ^*+*^ orientation in p3 and p7. ^1^H-NMR determined structures led to the D-13 mixture (**Table 1**) being developed to the point where a mixture of Mrz-derived mHABP AMA-1 **10022** + MSP-2 **24112** + EBA-175 **13790** + EBA-175 **24292** + SERA-5 **23426** displaying *gauche* ^*+*^ orientation in p3 (**Fig 1**) was added to the Spz-derived CSP-1 **25608** + CSP-1**32958** + TRAP **24254** mixture.

Trial D-13 involving the 19 *Aotus* immunised with the above mixture led to 6/19 monkeys (32%) producing high (though not as high as in previous trials) anti-SPz Ab-titres, and high (≥1:160) anti-Mrz titres in 5/19 different monkeys (~26.3%).

### Recognising reactivity with Mrz-derived recombinant fragments (WB)

The most relevant immunological information here was derived from WB analysis with the recombinant fragment. Two of the nineteen monkeys’ sera reacted with the MSP-2-rII fragment in mixture C-13 (as thoroughly demonstrated in previous monkey trials) [50], such reactivity being stronger with EBA-175-rI in another two monkeys out of the nineteen, suggesting clear genetic control by Ao HLA-DRβ1*0301 and Ao HLA-DRβ1*1406 for EBA-175 **24292**.

ALL monkeys immunised with Mrz-derived EBA-175 **13790** in group D-13 developed high reactivity with EBA-175 rI, and immunisation with AMA-1-derived **10022** (4313) induced very highly reactive antibodies against AMA-1 rI in 11 out of 19 of the immunised monkeys. Such immune responses suggested, as with Spz-derived mHABPs, that an immune response was being controlled by HLA-DR (as occurred with CSP-1 **25608** and **32958** in Spz and MSP-2 **24112** and EBA-175 **24292** Mrz-derived mHABPs) and a “universal” like or “promiscuous” immune response as occurred with TRAP **24254** and EBA-175 **13790**, the latter probably being induced by “universal epitopes” [46,47] able to bind to multiple HLA-DRβ1* alleles or displaying different TCR recognition [51] and activating multiple immune responses.

### *Aotus* challenge as another proof of concept

The appropriate fully-protective anti-malarial vaccine mixture was tested in this proof of concept trial when the monkeys participating in trial D-13 (one died before challenge) and the 6 control monkeys immunised with saline solution in Freund’s Adjuvant were challenged via intravenous inoculation of 500,000 infected erythrocytes freshly obtained from another *Aotus* monkey (previously infected with the highly virulent FVO strain) and their parasitaemia was assessed daily by sensitive accridine orange staining methods. **ALL** 6 control monkeys showed ~ 0.01% parasitaemia by day 4, reaching >5% by days 7–8; they were treated immediately [19,20]. Twelve of the 19 vaccines developed parasitaemia very similar to that of the control group and 4/19 (~21%) had no parasites in their blood during the time the experiment lasted (15 days). Another two of the 19 monkeys took two more days to control parasitaemia; however, they were not considered fully-protected according to our stringent definition of protection being absolute parasite absence in the blood during the time an experiment lasted. These fully-protected *Aotus* displayed a diversity of immune responses where two of them showed strong reactivity with the AMA-1 rI and EBA-175 rI fragments, another only with the EBA-175 rI fragment and another only with the AMA-1 rI fragment. This suggested these 2 proteins’ additive effect in protecting both monkeys. It should be remembered that SERA-5 (including **13790**) blot was not carried out since we were unable to express such protein as recombinant.

This data clearly supports the IMPIPS concept and suggests that more mHABPs have to be included to achieve complete full protective immunity against Mrz or the second line of defense.

### PPII_L_ relevance in IMPIPS conformation

**Fig 3:**
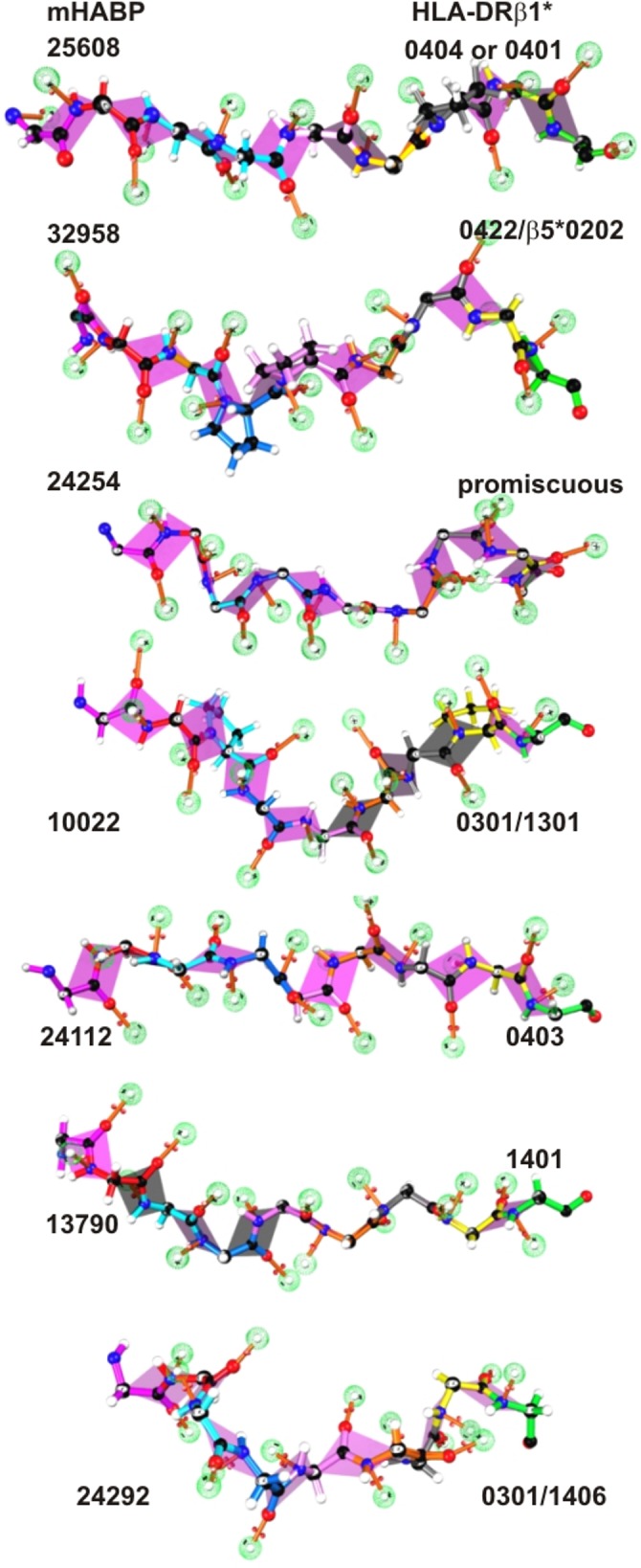
Spz- and Mrz-derived mHABP fragments binding to the HLA-DRβ1* PBR. Side view of mHABPs binding to the HLA-DRβ1* PBR (as assessed by ^1^H-NMR) displaying the residues according to the colour code: p1 (fuchsia), p2 (red), p3 (pale blue), p4 (dark blue), p5 (pink), p6 (orange), p7 (grey), p8 (yellow) and p9 (green). The dotted balls in light green represent the nonbonding free electron pairs able to establish H bonds with the HLA-DRβ1*PBR residues. The pink planes mark peptide bonds.

All VHLLAI and HIPI mHABPs which did not block, interfere, compete or suppress the immune response and were components of the mixtures presented here contained PPII_L_ structures (**Fig 1**) having ψ (135°±15°) and ɸ torsion angles (-95°±25°) [34,52] to fit into the HLA-DRβ1*PBR. Such PPII_L_ helixes are short amino acid sequences having 3–4 residues per turn, none stabilised by H-bonds, with turns where every third residue is stacked on top of the other [52–54], all amides being perpendicular to long helix axes [55], each residue being translated 3.12 A° (totally different to α-helixes having 3.6 residues per turn and each residue being translated 1.50 A°) [56] and a ~9.1 A° distance per pitch. These PPII_L_ helixes have aminoacid preferences in some positions [55,57] where branched residues, like Val and Ile, are not preferred due to their bulky conformation which occludes backbone solvation. Since HLA-DRβ1*PBR allows 9 residues to be accommodated, three PPII_L_ helixes provide the appropriate distance (26.5°±3.5° A°) to fit into Pockets 1 to 9 (**Fig 3**). This distance is not achieved when HABPs have α-helix (too short) [58], β-sheet (too long) [59], β-turn structures (having folded characteristic) [60], unless certain described chemical principles are accepted [61].

### Electron effects leading to PPII_L_ conformation in IMPIPS

**Fig 4:**
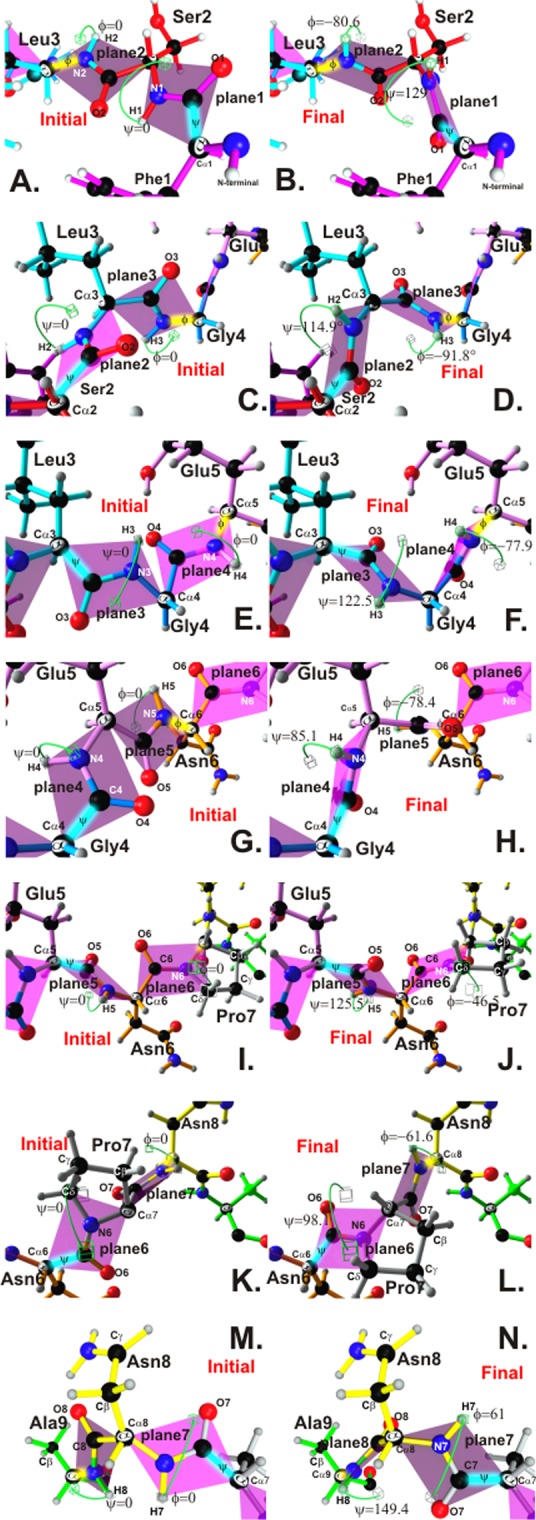
Comparison of initial and final peptide-bond planes for 25608.37. A, C, E, G, I, K and M. Green cubes indicate initial theoretical positions for **25068.37** regarding PBR residues forming Φ and ψ angles on planes 1 to 9. **B.**, **D.**, **F.**, **H.**, **J.**, **L.** and **N.** Final position of these angles reaching the lowest energy to avoid topochemical steric clashes, as measured for the lowest energy conformer, based on 3D structure obtained by ^1^H-NMR. Most angles were close to -93° ± 25° (Φ) and 134° ± 15° (Ψ), similar to PPII_L_. Minimisation values for atom clashes as determined by Ramachandran plot. HLA-DRβ1* classification according to experimental binding to purified molecules, binding motifs and binding registers is shown at the top on the right-hand side.

Analysing PPII_L_ conformation in IMPIPS for a better understanding of its fundamental role led to generalising that there would be a steric clash between H1 and O2 (**Fig 4A**, green cubes) if initial theoretical atom localisation and Ψ and Φ angle rotation in **25608.37** (**Fig 4A**) were compared in the lowest energy conformer measurement initial plane 1 and 2 position, taking Phe1 torsion angle Ψ = 0 and Leu3 Φ = 0 (as an example for all Ramachandran plot-based IMPIPS and Φ, Ψ, χ1, χ2 angles and ^1^H-NMR-determined distances); final 129° Ψ and -80.6° Φ rotation (**Fig 4B**) would thus have had to be induced to avoid such clash.

Initial steric repulsion of atoms located on planes 2 and 3 had intolerance to O2 with H3, having the scope of CH_3_ (**Fig 4C**) and the H3-O2 clash leading to 114.9° Ψ and -91.8 Φ final rotation (**Fig 4D**).

Initial angular proximity between planes 3 and 4 (**Fig 4E**) revealed H3-N4 repulsion in Φ. A 122.5° Ψ and -77.9° Φ rotation (**Fig 4F**) showed that minimum energy values located H3 far from the repulsion caused by H from one of the Leu3 methyl radicals and that O4 took up a distant position due to repulsion by Glu5 R O.

**Fig 4G** shows the initial position for an O4-O5 clash; a tolerable H4-R clash (Glu5) was noted when the minimum energy position was rotated 85.1° in Ψ and -78.4° in Φ (**Fig 4H**) due to R, whose antecedent to H4 is Gly4 whose small volume allowed other atoms to fit easily.

As Pro7 is cyclic (**Fig 4I**) it was involved in plane 6 (with Cα in one of vertices and with Cδ in another); its cyclic structure also produced very intolerant interactions regarding its neighbours since it behaved as a large, repulsion compact group, given its rigidity (**Fig 4J**). The O6-R (Asn6) clash was more accentuated and, due to its large volume including the complete R group (Pro7), it induced 125.5° Ψ and -46.5 Φ.

The initial part of **Fig 4K** shows clashes between H7-H8, N7-H8 and O7-C8 from planes 6 and 7, their minimum energy position being adopted on rotating Ψ 98.1° and Φ -61.6° (**Fig 4L**) whilst 149.4° Ψ and -61° Φ rotation was observed for planes 7 and 8 (initial **Fig 4M**—final **Fig 4N**).

All experimentally determined rotations in **25608.37** ranged from Ψ +115° to +130° and Φ -90° to -70°, these being very narrow limits for polyproline II-like structures [32]as this conformation is a key feature in high immunogenicity protection-inducing activity [33], as demonstrated below and previously shown for antigenic peptides determined by X-ray crystallography [34].

### Geometric and electron characteristics of IMPIPS residues: subatomic interactions

**Fig 5:**
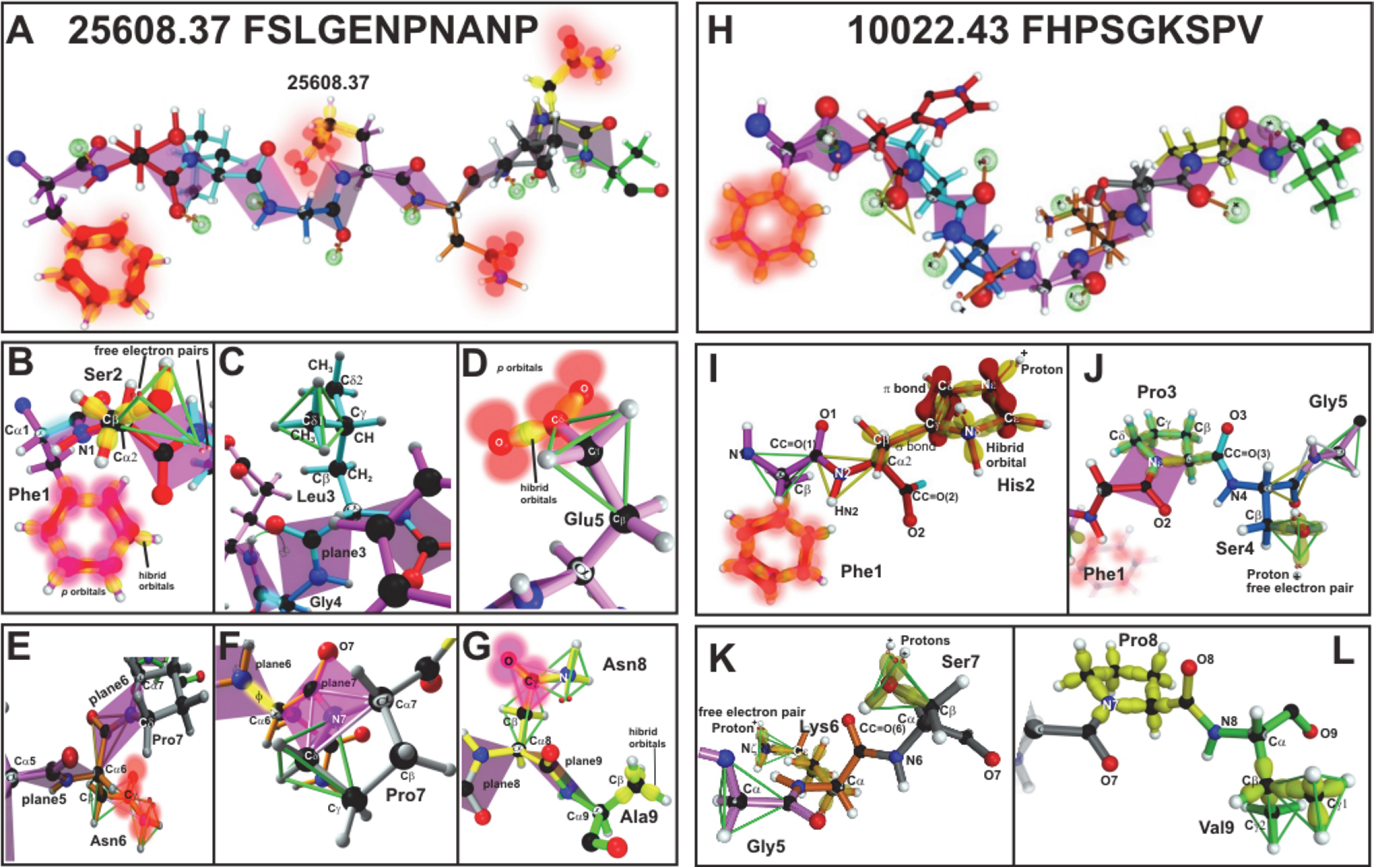
Steric-electron effects. 25608.37 (left panel) and 10022.43 (right panel) mHABP residues, displaying hybrid sigma (σ) orbitals (yellow), π and p orbitals perpendicular to them (red, blurred balloons). For **25608.37**: **A** and **B**. Phe1 displaying π resonance (red—bonds between p orbitals); Ser2 tetrahedron with the two free electron pairs (indicated) showing only the σ orbitals. **C**. Leu3 (green), showing the tetrahedron framing Cδ1 side-chain and orientation (pointing upwards), only Gly4 plane is shown. **D**. Glu5 (green), showing the tetrahedron framing Cɣ and trigon with Cδ and resonance between the two O and their corresponding Cδ from the side-chain (blurred red balloons); the electron charge is shown in blurred red orbitals. **E**. Asn 6 directed to Pocket 6, showing the tetrahedron, a trigon and the electron charge in blurred red. **F**. Pro 7 in grey and two trigons in green. **G**. Asn 8 directed toward the TCR with its corresponding p orbitals and its non-bonding free electron pair; Ala9 is also shown in green with a tetrahedron in the same colour. For **10022.43**: **H**. Phe 1 orientation, π resonance and its planes corresponding to peptide bonds. **I**. His2 showing the π resonance tiara. **J**. Pro3 cyclic structure with σ orbitals pointing upwards to contact the TCR, and also displaying the tetrahedron formed by the Ser4 side-chain pointing downwards. **K**. showing the tetrahedrons formed for Gly5 and Ser7 with its two free electron pairs. **L**. Pro 8 σ electrons pointing upwards and Val9, showing the two tetrahedrons framing Cδ1 and Cδ2 and their apolarity represented in σ-bonds.

Since molecules’ subatomic electron interactions and their implicit molecular geometry play a fundamental role in their fitting into the HLA-DRβ1 PBR, and therefore in their immunological performance, such characteristics were analysed regarding the best fitting Spz-derived CSP mHABP. This was **25608.37** (4383) (Phe1Ser2Leu3Gly4Glu5Asn6Pro7Asn8Ala9) (**Fig 5A**) which had high binding capacity (58%) to purified HLA-DRβ1*0401 molecules and had the characteristic binding motifs and binding registers for this allele family [45].

Therefore, classical aminoacid chemical structure analysis revealed that the first residue (Phe1, **Fig 5A** and **5B** pointing downwards) fitting into HLA-DRβ1*0401 Pocket 1 had 3 hybrid orbitals in each carbon (C), (**Fig 5,** yellow orbitals), forming 120° angles in trigonal planar geometry with the remaining valence electron occupying an orbital p_*z*_ (**Fig 5B**, blurred red orbitals). It overlapped another orbital p_*z*_ from its neighbouring C, thus forming Phe, Tyr, Trp aromatic π residues’ resonant bonds which were more functionality relevant than their own structure since the presence of π electrons appeared to be more important than backbone or side-chain conformation in aromatic residues to fit into MHCII PBR [62,63] (**Fig 5A**, red π bonds between p_*z*_ orbitals).

**Fig 6:**
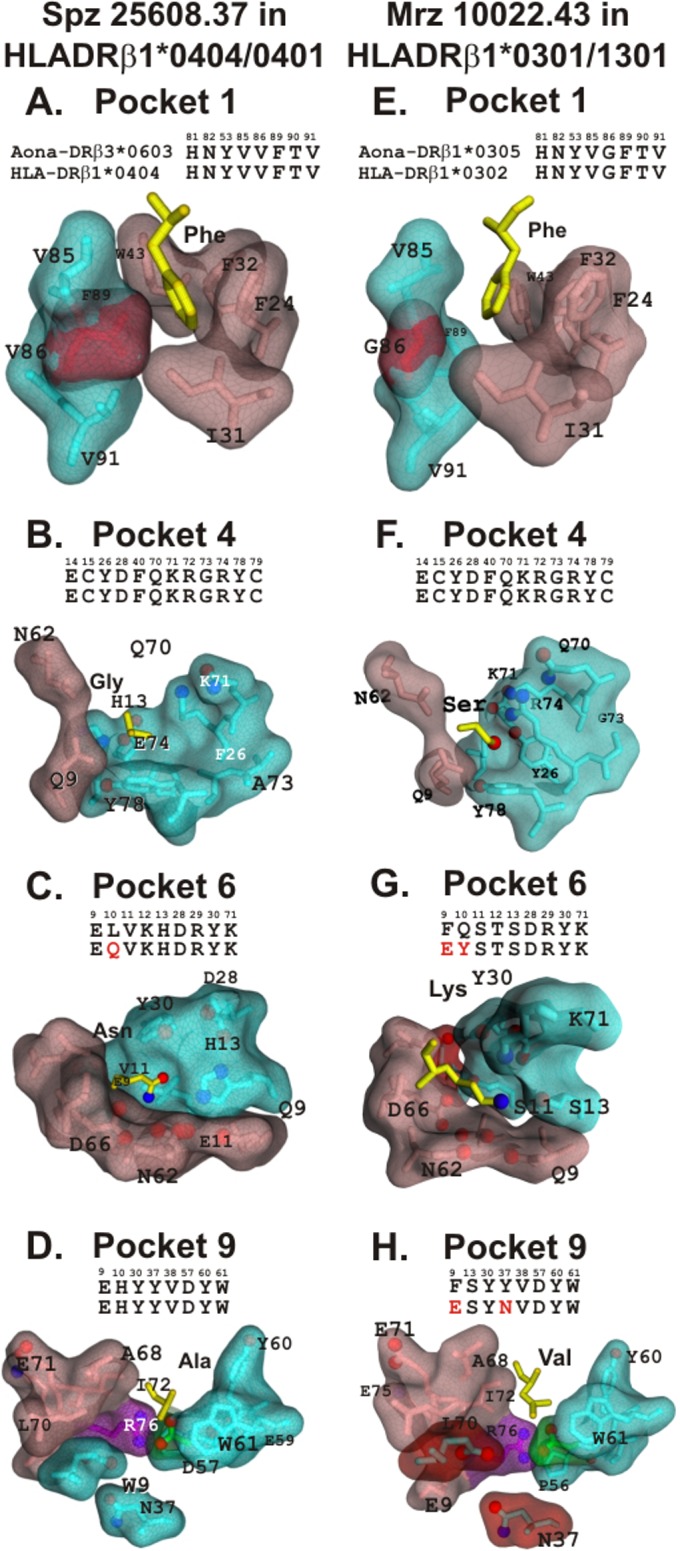
HLA-DRβ1* residues involved in Pocket 1, 4, 6, 9 formation and differences with *Aona* DRβ. Molecular surface of amino acids involved in Pocket 1, 4, 6 and 9 formation (α-chain in pink and β-chain in blue). Differences between HLADRβ1* and *Aona* DR are shown in red. The amino acids forming the pockets are shown on top of each one. **A.B.C.D.** Spz **25608.37** mHABP (yellow) fitting into HLADRβ1*0404/0401. The βV86 (red) is the dimorphic variant present in Pocket 1 in some HLA-DRβ1* molecules, showing that there were no differences between HLADRβ1*0422 and *Aona* DRβ3*0603 in this pocket. **E**. The same held true for HLADRβ1*0301 and *Aona* DRβ1*0305 in this pocket. **G**. *Aona* DRβ1*0305 Fβ9E (in red) replacement in Pocket 6 was located far away from Pocket 6 side wall; it therefore had no impact on mHABP binding when compared to HLADRβ1*0301. **H.** The 2 replacements observed between *Aona* DRβ1*0305, HLADRβ1*0301 (Fβ9E and Yβ37N, both in red) had no impact in Pocket 9 since they were located far away from this pocket’s floor. These mHABPs were thus very similar and could be used for human immunisations without any further modifications.

The highly resonant and hydrophobic structure of aromatic residues (Phe in p1) enabled fitting into highly hydrophobic HLA-DRβ1* Pocket 1 formed by an array of aromatic and apolar residues αF24, αF26, αI31, αF32, αW43 and αF54 in the HLA-DRβ1* α-chain and βY83, βV85, βG86, βF89 in its β-chain [62] (**Fig 6A**). Phe, Tyr and Trp preference for Pocket 1 could be attributed to the aromatic–aromatic electrostatic interaction [64] with this pocket’s aromatic residues. This was particularly true for side wall, evolutionarily conserved, αW43 [65]; this Pocket 1 space was only limited by the size of the dimorphic Vβ86G variant, rendering this pocket smaller, thereby preferring large apolar residues like Leu, Ile and Val, but tolerating also Phe, but not Tyr, nor Trp (**Fig 6A** and **6E**). This dimorphic variant occurs in all *Aotus* allelic families [36,66].

Phe and Tyr are common in β-sheets and PPII_L_ conformations due to favourable electrostatic interactions with neighbouring structures [67]; thus it is not unusual to find such residues at the beginning of mHABP PBR binding residues or at the C terminal of mHABP upwardly oriented residues to contact the TCR, perhaps as a consequence of cation- π interaction [68]. Due to strong π electrostatic interactions, Phe, Tyr or Trp (less frequently) are the preferred amino acids in Pocket 1 interactions [69–71].

Ser geometry (**Fig 5B**) regarding p2 and pointing away from the PBR consisted of 2 tetrahedrons (one containing Cβ and another projecting H from O in inclined position, occupying one of the tetrahedron’s vertices) while 2 pairs of non-bonding free electron pairs from O were situated in the other 2 vertices. Together with Thr, these two small amino acid hydroxyl groups function as nucleophiles on enzymatic reactions [62]; these 2 non-bonding free electron pairs (**Fig 3** green celosias) may thus be very relevant in H-bond formation and interaction with TCR CDR1α atoms.

Also pointing away from the PBR, all Leu3 carbons (C) interacted by means of single σ-bonds where Leu3 geometry began with the side-chain and each CH3 (Cδ1 and Cδ2) was framed within tetrahedrons (**Fig 5C**, in green) where 3 H and one C (from CγH) were located in its vertices. The Cα, Cβ and Cγ geometry of this apolar aliphatic chain was determined by *sp* ^*3*^ hybridisation of each tetrahedron C atom, constituted by sigma (σ) bonds, the *gauche* ^*+*^ orientation of these p3 and p7 residues being critical in protective immunity induction [21] since these residues could be strongly interacting with antigen-specific somatically-generated TCR CDR3α and CDR3β regions. This has been demonstrated in the HLA-DRβ1*0401-HA-TCR complex [31,72] as well as in HIPI **24112** when bound to HLA-DRβ1*0422 and recognised by the TCR [50].

Position 4 for fitting into Pocket 4 in this mHABPs was occupied by Gly (**Fig 6B**) and, despite this pocket 4 being extremely important in some alleles (HLA-DRβ1*03 and other HLA-DRβ1*04 variants), this amino acid’s small size did not permit an in-depth analysis in this mHABP.

Pointing away from the PBR, Cβ and Cγ in Glu5 (**Fig 5D**) were circumscribed in tetrahedrons (Cγ in green, **Fig 5D**) though Cδ was contained in a trigon containing Cγ in its vertices, both O having 6 valence electrons and *p* orbitals in resonance to balance the electron charge resulting from O hybridisation similar to that of C (*sp* ^*3*^). The electron charge (displayed in blurred red orbitals in **Fig 5D**) was thereby distributed in its Cγ and two O reactive atoms, having two pairs of non-bonding free electron pairs both of which could have been establishing H-bonds with some other atoms from the TCR CDR3α region.

Cβ in *sp* ^*3*^ hybridisation in Asn in Pocket 6 was contained in a tetrahedron whose vertices were occupied by Cα, HCβ1, HCβ2 and Cγ, while Cγ was contained in a trigon in *sp* ^*2*^ hybridisation due to neighboring atoms where N and O occupied the planar trigon’s vertices (**Fig 5E**). The O in this trigon also belonged to another trigon whose vertices could be represented by each 2 non-bonding free electron pair and Cγ; as O has 6 valence electrons, two of them must be located in orbital p_z_ and this charge must be shared by a π bond formed with the p orbital from Cγ (**Fig 5E**). Pocket 6 in HLA-DRβ1*0401 was formed by an array of negatively-charged residues, where conserved residues αE11, αD66, αQ9, αN62, αN69 and βE9, βQ10, βD28, βK12, βH13, βR29 and βK71 (**Fig 6C**) generated a small pocket where Asn 6 fit and its non-bonding electron pair interacted with βH13 to stabilise binding to this pocket. Pocket 6 in all MHCII molecules contains an unexpected αE11 and αD66 carboxylic pair which are protonated and stabilised by a network of H-bonds which, together with the other negatively-charged residues, could partially explain the absence of D or E in this position in any peptide fitting into this MHCII pocket.

Two trigons were present on the Pro7 plane in the region pointing away from the PBR; their side chain cyclised ring (Φ = -65°±15°) [73] was upwardly projected and towards the right (**Fig 5E** and **5F**) with some atoms pointing upwards to interact with some residues in the TCR CDR3β region. Pro7 conferred rigidity on this part of the molecule since Cα and Cδ contiguous to N7 were plane 7 components. Moving, shifting or turning this plane would thus contemplate more steric hindrance since it requires mobilisation of the whole radical group.

Asn, in p8 pointing away from the PBR, had similar geometry to that described for Asn6 as its O donor could be making contact with TCR CDR3β residues due to its *p* orbitals and its non-bonding free electron pair (**Fig 5G**). It is well-known that Asn, Gln and His side chains benefit from flipping on their planar groups for establishing H-bonds [74].

Ala geometry in Pocket 9 involved a hybridisation where its Cβ was circumscribed in a tetrahedron (**Fig 5G**) with σ orbitals. The small size of Pocket 9 and its hydrophobicity could be partly attributed to the salt bridge formed by β57D-α76R (**Fig 6D** βD57 in green and αR76 in purple) and apolar residues like αF71, αA68, αL70, βW9 and βW61 (**Fig 6D**) limiting Pocket 9 size and polarity allowing only small apolar residues to fit. βD57 thus enabled the fitting of small apolar residues like Ala9 due to this size limitation in *Aona* HLA-DRβ3*0603 and DRβ*4704 GA linages, corresponding exactly to HLA-DRβ1*0404, 0401 and 0403. In non-βD57 alleles, having βS57, βT57 or βV57 variants, αR76 allowed the fitting of large polar or apolar residues, depending on the variants in 9β (like βE9, βK9 or βW9), since the βD57-αR76 salt bridge was not formed, leaving the two α76R non-bonding electron pairs free to interact with the p9 residue side chain. These two electrostatic characteristics determined residues polar preference for fitting into p9, the most representative being HLA-DRβ1*0405, 0801 and 0805 binding peptides with E, D or Q in p9.

Since no differences have been found in these pockets between *Aona* DRβ3*0603 and W4704 GA and HLA-DRβ1*0404, 0401, 0403 and the most common human HLA-DRβ1*04 alleles [36,40,75], these VHLLA- and HIPI-inducing mHABPs inducing protection in *Aotus* monkeys against experimental challenge could be used for human vaccination against *P*. *falciparum* malaria without any further modification.

Another example involving Mrz-derived AMA-1 **10022.43** (4313) (Phe1His2Pro3Ser4Gly5Lys6Ser7Pro8Val9) (**Fig 5H**), having high experimental binding capacity to HLA-BRβ1*0301 (52%) purified molecules and being predicted to bind to HLA-DRβ1*13 and β5*0101 allelic families (data not shown), displayed the binding motifs and binding registers characteristic of these molecules, induced VHLLAI titres as assessed by IFA, recognised the AMA-1 rI fragment (38.5 kDa) by WB and led to full protective immunity against experimental challenge in ~10% of immunised *Aotus*. Electron and topochemical characteristics were also analysed; the Phe1 residue was the same as in **25608.37**, fitting into Pocket 1. The only difference concerned the β86G dimorphic variant in HLA-BRβ1*0302 in this pocket, even though aromatic residue like Phe were preferred by this allele in this pocket.

Regarding p2 (**Fig 5H**), C4 from His bound to N3 by a simple covalent bond, by a double bond to C5ɣ and by single bond to H, all of them having trigonal planar geometry. Three σ bonds were established, one binding to the C2 hybrid orbital, another to C5ɣ hybrid orbital and the other to the H *s* orbital, leaving one non-bonding free electron pair solvated at the vertex of the C2, C5ɣ tetrahedrons. These two free nonbonding electron pairs were able to interact with two protons, one on top and the other at the bottom of the His (**Fig 5I**) tiara, allowing the formation of a π resonant structure able to interact with the TCR CDR1α.

The unique structure for Pro in p3 can be seen in **Fig 5J** where the C6(δ) atom from the aliphatic chain covalently binds to its N backbone atom making it cyclic, having consequences in the protein chain: -65°±15° in Pro ɸ and Cδ sterically restricted the preceding ψ mobility [56,76]. If Pro ɸ were -62.4° or less, this residue would have been orientated upwards (**Fig 5H** and **5J** and **Fig 1**) and if ɸ were -75° or more it would have pointed downwards. Pro had *gauche* ^*+*^ orientation in p3 in this mHABP thereby confirming the relevance of this residue’s orientation in full protective immunity induction, perhaps by its contact with the somatically-generated, antigen-specific TCR CDR3β region.

Concerning p4, Ser stereo-electron characteristics have previously been described for **25608** as not being different to that of **10022,** having similar ɸ and ψ angles. Ser is the second most frequent residue binding to Pocket 4 in HLA-DRβ1*03 which, in *Aotus* monkeys, corresponds to the *Aona* DRβ1*0305 GA family which is almost identical to its human counterpart, having ~21% phenotype frequency [36,66].

Gly had the same basic structure in p5 and it is very often found in—p1 or—p2, preceding PPII_L_ structures [77], as in **10022** (**Fig 5J**) or **25608,** where it preceded the second PPII_L_ helix located in these two mHABPs’ C-terminal (**Fig 1**) or the PPII_L_ region in **13790.45** confirming this residues preference for these positions.

Regarding Lys in Pocket 6, the four N orbital hybrids pointed towards the vertices of a tetrahedron; one contained a non-bonding free electron pair which interacted with a proton, thereby establishing a dynamic bond (**Fig 5K**). Even when this N fitted into a tetrahedron, it had trigonal pyramidal geometry. The other C from Lys was framed within tetrahedrons and had sp3 hybridisation when interacting with four neighbouring atoms. This Lys bound to Pocket 6 where Eβ9F and Yβ10Q amino acid replacements were found in the *Aona* DRβ1*0305 GA allele family, the former located in a very distant region of Pocket 6 (**Fig 6G**, surface in red) and the other not being involved in this pocket’s conformation. Such replacement did not make any difference between *Aotus* and human HLA-DRβ1* Pocket 6, such data being confirmed by analysis of the three multipolar moments of the electrostatic field by quantum chemistry [78].

The stereochemical characteristics of Ser in p7 have been previously described, 2 protons being ready to interact with the TCR CDR3β region. This residue displayed the same *gauche* ^*+*^ rotamer orientation in **10022** as occurs in all VHLLAI and HIPI mHABPs (**Fig 5K**).

Topochemical and electron characteristics have been described for Pro in p8 (**Fig 5L**) and, as beforehand, a ɸ-62.49° torsion angle orients this residue upwards to make contract with the TCR, probably with CDR3β and/or CDR1β.

Val geometry in Pocket 9 consists of tetrahedrons, each circumscribing Cα, Cβ, Cγ1 and Cγ2 where the 3H from Cγ1 and Cγ2 were located in their vertices (**Fig 5L**) to fit into HLA-DRβ1*0301 Pocket 9. There were two amino acid replacements in this pocket in the *Aona* DRβ1*0305 GA allele: the previously mentioned Eβ9F shared Pocket 6 and Nβ37Y located on the floor of this pocket (none limiting Pocket 9 size or its electron characteristics as previously mentioned for **25608.37** and as shown by quantum chemistry) [78].

VHLLAI and HIPI mHABPs found to be immunogenic and protection-inducing in the *Aotus* monkey population (i.e. **10022** fitting into *Aona* DRβ1*0305GA, corresponding to human HLA-DRβ1*03, 13 or β5*0101 characteristics [36,40,75]) could also be used for human vaccination against *P*. *falciparum* malaria without any further modification.

### mHABP Φ and Ψ angles for residues in PPII_L_ conformation allow IMPIPS fitting into HLA-DRβ1* PBR

The PPII_L_ conformation (based on our H-MNR studies of ~100 mHABP structures) determines not only the appropriate distance for fitting into HLA-DRβ1* PBR Pockets 1 to 9 but also the appropriate orientation of the N and O peptide bond nonbonding free electron pairs (**Fig 3**, green celosia) to firmly establish the H-bonds or Van der Waals (vdW) interactions with corresponding HLA-DRβ1* anchoring side-chain atoms.

Spz CSP-derived **25608.37** and Mrz AMA-1 **10022.43** best fit conformers (i.e. number after dot) and their Φ (-95± 25°) and Ψ angles (+135±15°) we described here as examples of structurally dependent characteristics, some limited angular deviation occurring in the region fitting into their HLA-DRβ1*PBR having polyproline II left-hand (PPII_L_) helixes (highlighted in gray in **Fig 1**).

The paper shows that almost all VHLLAI and HIPI mHABPs contained one or two PPII_L_ helixes, sometimes spaced by Gly to allow a proper fit into their corresponding HLA-DRβ1* PBR [32,33]; however, some mHABPs also contained α_L_ or β-turn regions (**Fig 1**, highlighted in pale blue or yellow, respectively). This tantalising problem was resolved when a low-energy pathway converting PPII_L_ into α-helixes via β-turn formation, without breaking the H-bonds, and introducing an n→π* stabilising interaction (or hyper-conjugation [79–81]) were proposed to avoid steric clashes (Lennard-Jones potential). Such transition could occur in antigen-presenting cells’ phagolysosomal acidic environment and enable their appropriate modification to allow loading these mHABPs into HLA-DRβ1* molecules’ PBR. This hyper-conjugation [79–81] could have occurred in **24254.31**, mainly containing a type III β-turn (**Fig 1**, highlighted in yellow) and **23426.35** (**Fig 1**, highlighted in gray) containing a symmetric PPII (sPPII) region (Φ -145°±15° and Ψ 60°±15°), the mirror image of PPII_L_ conformation [82].

The 3D structure of the IMPIPS included in this manuscript was determined by ^1^H-NMR; besides the PPII_L_ helixes, other structures in these mHABP are also worth mentioning (**Fig 1**). The PBR residues in **32958.2** were followed by a β-turn type I while a β-turn distorted III’ preceded them in **10022.43; 24112.39** had a distorted β-turn type III’ at the N terminus and another classic β-turn type III' formed part of the N terminus of the PBR. There was a α_L_-helix region in **24292**.**12** and a β-turn type V preceding the PPII_L_ region in **23426.35**. A distorted β-turn type I followed the PBR in **13790.46.**

PPII_L_ helixes are thus the structures best displaying the binding motifs, binding registers and steric-electron topochemical characteristics to fit into the HLA-DRβ1* PBR to properly present IMPIPS to the TCR.

### mHABP backbone atom interaction with MHCII side-chain atoms

**Fig 7:**
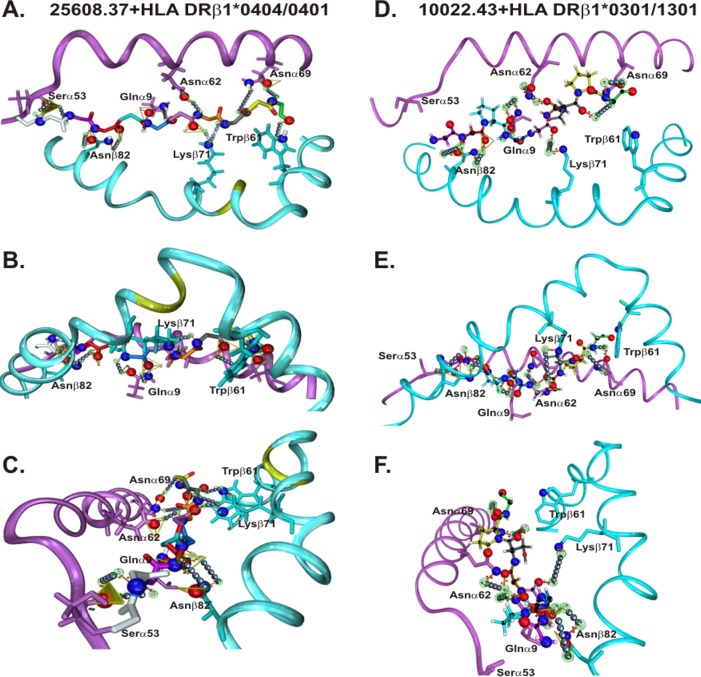
H-bonds established between HLA-DRβ1*0404/0401 with 25608.37 and HLA-DRβ1*0301 with 10022. A.D. Front view B.E. Top view C. F. Side view. Left panel: **25608.37**—HLA-DRβ1*0404/0401. Right-hand Panel: **10022.43**—HLA-DRβ1*0301. Their atomic distances are given in **S1 Fig**.

NB: 3D structures were determined by two very different methods: ^1^H-NMR (in solution) for **25608.37** and **10022.43** as the prototype mHABPs and X-ray crystallography for HLA-DRβ1*0401 (PDB code 1J8H) [72] and HLA-DRβ1*0302 (PDB code 1A6A) [25] as templates for docking studies (**Fig 7**). Some differences could thus have occurred due to the different methodologies used, i.e. HLA-DRβ1*0404/0401-like (Aona DRβ*W4704 GA family) and HLA-DRβ1*0302 (Aona HLA-DRβ1*0305 GA allelic family). HLA-DRβ1*-like structures were modified by molecular dynamics based on the few differences with *Aotus* [36,40] (golden in HLA-DRβ1* β-chain blue ribbon in **Fig 7**), no further changes or refinement being needed.

Our group has previously shown (**S1 Fig**) that all ^1^H-NMR obtained structures spontaneously formed H-bonds or vdW interactions when superimposed onto HLA-DRβ1* platforms. Spz-derived VHLLAI **25608.37** thus established 7 spontaneously formed H-bonds (≤ 3.5 Å) and 3 vdW interactions (≥3.5Å ≤ 4.5Å) when superimposed onto modified HLA-DRβ1*0422, involving Qα9, Sα53, Nα62, N α69, Wβ61 and Nβ82 (**Fig 7A, 7B** and **7C**) and mHABP **32958.2** formed 7 H-bonds and 2 vdW interactions when superimposed onto HLA-DRβ1*0422, involving similar residues but also Kβ71. When **24254.31** was superimposed onto HLA-DRβ1*0401 it formed 6 H-bonds and 3 vdW interactions with the α- and β-chain canonical residues from this allele, and formed 6 H-bonds and 5 vdW with HLA-DRβ1*0403.

By the same token, Mrz-derived HIPI **10022.43** spontaneously formed 5 H-bonds and 3 vdW interactions with HLA-DRβ1*0302 (**Fig 7D, 7E** and **7F**) while **24112.39** had 5 H-bonds and 2 vdW interactions with HLA-DRβ1*0403. **13790.46** had 7 H-bonds and 2 vdW interactions with HLA-DRβ1*0401 and **23426.35** established 6 H-bonds and 3 vdW with HLA-DRβ1*0403.

**Fig 8:**
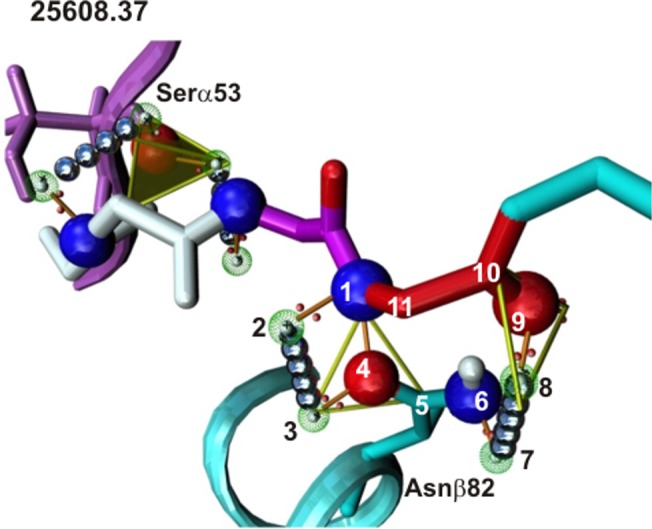
Ring structure formation in mHABP 25608.37. H-bonds or vdW interactions between Ser2 **25608.37** N and O backbone atoms and HLA-DRβ1*0404/0401 conserved Nβ82 side-chain atoms; this led to establishing ring structures involving the 11 atoms shown here. These 9–11 ring atoms structures were also established with Qα9, Nα62 and Nα69, thereby stabilising IMPIPS binding to the HLA-DRβ1* PBR.

This confirmed previous results which had shown that HLA-DRβ1* and *Aona* DRβ1* conserved Nβ82, Qα9, Nα69 and Nα62 formed 9–11 atom ring structures (**Fig 8**) with VHLLAI and HIPI backbone atoms to establish the necessary 9–11 canonical H-bonds or vdW interactions (**Fig 7**) for properly anchoring mHABPs to the MHCII PBR. The foregoing, together with the bonds established by Sα53 with—p1, the variable (T, R, N or K) β71 residues and conserved Wβ61 (thereby establishing one H-bond each with the peptide’s backbone) might lead to very strong, stable pMHCII complex formation to be presented to the TCR for inducing an appropriate immune response, as previously shown for antigenic peptides (**Figs 7** and **8** and Supporting Information (**S1 Fig**)).

### Topochemical localisation and electron characteristics in upwardly-oriented mHABP residues

Once the structural topochemical and electron characteristics of VHLLAI and HIPI mHABP binding to HLA-DRβ1* and Aona DRβ1* had been analysed, the MHC-peptide complex to be presented to the TCR to form the appropriate MHCII-mHABP-TCR tri-molecular complex was subjected to similar analysis regarding the proper activation of the immune response.

The TCR adopts a canonical diagonal orientation to conform a stable MHCII-p-TCR complex [28–31], thereby limiting TCR specificity and signalling [83]. The stereo-electron characteristics of residues in mHABPs pointing away from the PBR (p2, p3, p5, p7 and p8) and theoretically contacting the TCR were thus analysed in detail, based on all monkey trial data and ^1^H-NMR structural analysis.

**Fig 9:**
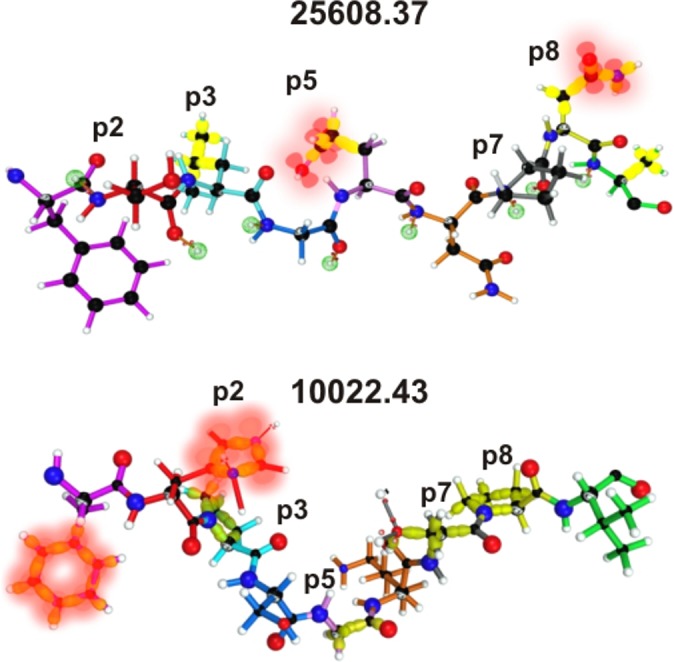
mHABP electron density. Side-chain atoms for p2, p3, p5, p7 and p8 in 25608.37 and 10022.43. The figure shows the side-chains for upwardly-oriented residues pointing to TCR-contacting residues. Polar amino acids present in p2, p5 and p8, displaying their non-bonding free electron pairs and π orbital surfaces shown in blurred red while σ orbitals for apolar ones present in p3 and p7 are shown by yellow surfaces. The φ angles (≤ -64°) in p7P (**25608.37**), p5P (**32958.2**), p7P (**24254.31**), p3P and p8P (**10022.43**) oriented this residue to make contact with the TCR.

It is worth noting that All IMPIPS had specific electron density regarding upwardly-pointing or TCR-contacting residues, i.e. all had charged residues in p2 with π orbitals (His) or non-bonding free electron pairs (Ser, Asn, Thr, Gln); likewise, all were aliphatic (Leu, Val, Met, Ala) or small apolar (Gly and Pro) residues in p3. All p5 residues were charged with non-bonding free electron pairs or upwardly-pointing Pro, similar to p8 residues having the same characteristics or π electro-resonant structures. No relevant electrostatic preference was identified in p7 but they display a *gauche* ^*+*^ orientation (**Fig 1**). These striking differences suggested that TCR CDR-contacting residues had a specific electron and rotamer orientation preferences (**Fig 9** and **Fig 1**).

### Rotamer orientation regarding mHABPs in upwardly-oriented TCR contacting residues

When analysing the rotamer orientation of IMPIPS that did not induce competition or interference with or blocking or suppression of other mHABPs when mixed, it was found that all their p3 aminoacid dihedral χ1 angles had *gauche* ^+^ orientation (**Fig 1**, highlighted in purple) (-174°* to -24.7°) [74,78,84–87]. It was also noted that all these IMPIPS mHABP p5 χ2 had *gauche* ^+^ orientation which was not observed in any other mHABP inducing interference, blocking, competing or suppressing the mHABP mixtures studied so far. Similarly, the p7χ1 angles had *gauche* ^+^ orientation (**Fig 1**, purple) while most simply antigenic or just immunogenic, non-protection inducing mHABPs p7 side-chains had *gauche* ^+^, *gauche* ^-^ or *trans* orientation at random.

The forgoing results suggest that p3χ1, p5χ2 and p7χ1 angles in *gauche* ^+^ orientation and polarity in p2 (S, N, H, T) and p8 (N, S, D), all displaying non-bonding free electron pairs or π resonant structures (F), are the key stereo-electron topochemical features for TCR contact and an appropriate mixture composition for a complete fully protective antimalarial vaccine, thereby ensuring that a VHALLAI- and HIPI-induced immune response is properly activated.

It has been elegantly demonstrated that conformational changes [88,89] and flexibility in the TCR, such as hinge modification or bending in the somatically-generated, hypervariable CDR3β loop (which is highly relevant in antigen specificity) and/or loop remodelling in CDR3α [90] forming the TCR V3 region [91] enable the complex mechanism of TCR structural modification to facilitate rapid sampling of pMHCII complexes by the TCR [92]. It may thus be speculated that such difference in polarity might account for specificity or promiscuity occurring in an immune response [51].

It was noted that mHABP **25608.37**, **32958.2**, **24112.39**, **24292.12**, **10022.43** and **23426.35** did not block, interfere, suppress or compete in mixtures; they displayed specific reactivity with their corresponding recombinant proteins and were under stringent and specific HLA-DRβ1* genetic control. Moreover, canonical p3χ1, p5χ2, p7χ1 *gauche* ^*+*^ orientation in all of them was apolar, aromatic or involved a positively-charged guanidinium residue (R7 in **24112**), while the p7 residue was polar (D) in those displaying universal reactivity, like **24254.46** and **13790.46**. This suggested that such promiscuity probably had more to do with this residue’s polarity for contacting the TCR than promiscuity in HLA-DRβ1* PBR binding (or both), a hypothesis currently being under study in our institute.

### IMPIPS’ biological relevance

The large number of negative and very disappointing biologically-derived human vaccine trials during the last four decades and countless animal tests providing protection in some immuno-genetically homogeneous (congenic) animal strains that have yielded negative results when tested in different ones, or in outbred populations or in some other species has meant that a necessarily different approach has had to be adopted regarding vaccine development.

Our group decided to tackle the problem from a chemical point of view ~30 years ago [10,11,18–20,35] in the search for a logical and rational methodology for vaccine development which now allows us to introduce the IMPIPS concept, its methodology and results, based on the wide-ranging possibilities provided by chemistry and physics, correlated with the biological and immunological function tools analysed here.

Advantage was thus taken of chemical reactions’ exquisite specificity, i.e. receptor-ligand interaction between host cells and the parasite molecules, HLA-DRβ1* binding capacity, 3D structure determination and many more chemical reactions that can be analysed at the single atom level. Other benefits provided by chemistry, such as reproducibility, low lost, stability, etc., make chemically-synthesised vaccines the answer to matters concerned with human health and welfare.

Appropriate target selection in choosing *P*. *falciparum* protein conserved high activity binding peptides (cHABPs—some of whose biological functions have been identified at 3D level [46,93,94]) and the rules or principles for converting immunologically silent cHABPs into highly immunogenic and protection-inducing mHABPs [18–20] has allowed us to contextualise the IMPIPS concept with its own methodology, principles and results. Such approach led to identifying more than 100 Mrz-derived mHABPs inducing HIPI against experimental challenge when individually used as immunogens and more than 50 Spz-derived VHLLAI mHABPs to be included as components of a fully protective complete antimalarial vaccine [18–20,35].

A tantalising problem arises in vaccine development when vaccines are mixed or, in our case, when mHABPs were mixed their individual immunological activity disappeared or dropped dramatically, suggesting suppression, blocking, interference or competition [41–43].

A very recent solution to this problem was presented when PPII_L_ structures [32,33] were found in mHABP 3D structure (determined by ^1^H-NMR) and that the side-chains of residues in p3 and p7 of amino acids fitting into HLA-DRβ1* PBR had *gauche* ^+^ orientation in their χ1 angles [21].

Further monkey trials were thus performed to establish the principles or rules for an effective antimalarial vaccine mixture.

Since our vaccine components are very relevant regarding many of a parasite’s biological functions, immunisation with corresponding mHABPs could induce blocking a parasite’s biological functions, their destruction or even death. A brief description of such biological functions is thus given below.

Sera from monkeys developing anti-**25608** antibodies [35], induced by modified CSP- 4383 ^68^(NS**R**S**L**G**E**NDDGNNEDNEKLR)^87^ located in region I (RI); an 11 kDa rCSP N terminal fragment or rI where RI is located was also recognised by WB. This RI contains the RxLxE Plasmodium export element (PEXEL) motif mediating CSP entry to hepatocyte cytoplasm and кB nuclear gene activation for inducing Spz proliferation and differentiation into Mrz. cHABP 4383 was also located fifteen residues upstream the ^102^(KLKQP)^106^ amino acid sequence used to bind to glucosamine glycan (GAG) and heparan sulphate (HS) moieties on hepatocyte membrane. This is a very relevant region since a 10-day delay in disease patency has been shown when RI was knocked out in Spz. Anti-**25608** antibodies produced by *Aotus* monkeys [45] could thus be blocking all these essential functions for Spz survival.

mHABP **32958** was derived from CSP-1 cHABP 4388 [35] ^286^(GNGQGHN**M**PNDOP**NRN**V**D**ENA)^305^, located five residues downstream the tandem repeat region in RII, containing the 19 aa-long (residues 338–355) thrombospondin-related (TSR) cell adhesion domain binding to heparan sulphate proteoglycans (HSPG) on hepatic cells. Anti-**32958** sera reacted by WB with the 16 kDa CSP1-rII fragment, suggesting that these antibodies could be sterically hindering RII activity (**Fig 2**).

TRAP cHABP 3347 ^541^(YAGEPAPFDETLGEE)^555^, located in this microneme protein’s C-terminal region which is translocated to the Spz membrane, lay 17 residues upstream the amino acid sequence containing the aldose’s binding site for the motor machinery mediating Spz gliding and cell traversal activity and also two residues upstream the acidic amino acids required for parasite survival. Besides reacting by IFA, anti-**24254** mHABP (derived from 3347) also reacted by WB with the 12 kDa TRAP rII fragment [49], suggesting that these antibodies could be interfering in some way with this protein’s translocation to Spz membrane, Spz gliding motility, and traversal activity which are extremely important functions for Spz survival [95–97].

Spz-derived IMPIPS could be blocking Spz gliding motility, cell traversal activity and hepatocyte invasion mediated by their corresponding cHABPs, rendering them excellent epitopes to be included as components of the **first line of defence** for a fully-protective antimalarial vaccine.

Anti-Mrz antibodies directed against mHABP **10022** produced by monkeys carrying the HLA-DRβ1*0301 allele could have been blocking the trough or niche formed (via H-bonds) by AMA-1-derived cHABP 4313 ^134^(DAEVAGTQYRLPSGKCPVFG)^153^, with cHABP 4325, where a still unrecognised RBC receptor bound, impeding firm, AMA-1-region-mediated, Mrz attachment and reorientation to initiate penetration of RBC [18–20]. It may well also impede resealing of the RBC membrane at the posterior end during parasitophorous membrane formation. Blocking some of these functions makes this mHABP an excellent candidate to be included as a component of a multi-antigen vaccine.

Antibodies produced in *Aotus* with HLA-DRβ1*0403 against mHABP **24112** could have been blocking MSP-2 cHABP 4044 ^21^(KNESKYSNTFINNAYNMSIR)^40^ located in this abundant Mrz membrane protein N-terminus one residue upstream of this **i**ntrinsically **d**isordered **p**rotein (IDP) structure [98], enabling invasion of RBC and binding to RBC phosphatidylinositol (PI) moieties [18–20,99].

EBA-175 played a pivotal role in rolling over and RBC invasion, being enzymatically processed into several fragments. 1758 ^80^(KSYGTPDNIDKNMSLIHKHN)^99^ was located in the N-terminal region named RI and 1815 ^1220^(YTNQNINISQERDLQKHGFH)^1239^ in the C-terminal portion containing regions IV—V which are involved in microneme trafficking to the Mrz membrane, inducing neutralising Abs against multiple *P*. *falciparum* strains. Anti **13790** and **24292** mHABP antibodies could thus have been blocking such processes [18].

cHABP 6754 ^741^(YKKVQNLCGDDTADHAVNIVG)^760^ contained two of the amino acids involved in the non-canonical triad of the SERA-5 protein enzymatic site (H755 and A756). Anti-**23426** produced in *Aotus* typed as HLA-DRβ1*0403/0101 could have been blocking this critical enzymatic site [18–20] which is very relevant in Mrz egress from infected RBC [93,100,101].

Chemically synthesised Mrz-derived HIPIs mHABPs were thus seen to be inducing sterile immunity against very different functionally-relevant targets, supporting our functional approach and making them undoubtedly basic components in a second line of defence (Mrz), to cover different HLA-DRβ1* genetic backgrounds.

Deeply analysed stereochemical and topological mHABP characteristics have a great impact on multi-antigen, multistage, complete, fully protective vaccine development since all mHABPs inducing VHLLAI and HIPI responses in IMPIPS mixtures had the appropriate distance to fit into HLA-DRβ1* Pockets 1 to 9 (**Fig 3**). Most mHABPs were or contained PPII_L_-like conformation. ALL peptide bonds forming N and O atoms had appropriate stereo-electron orientation in their backbone to establish H-bonds or vdW interactions with their corresponding HLA-DRβ1* side-chain atoms (**Fig 7A, 7B** and **7C** for **25608.37** with HLA-DRβ1*0404/0401 and **Fig 7D, 7E** and **7F** for **10022.43** with HLA-DRβ1*0301/1301 as example). The residue in p2 for ALL mHABPs having side-chains pointing away from the PBR were polar, displayed non-bonding electron pairs or π forming amino acids while residues in p3 were aliphatic. All but one in p5 were charged or had upwardly oriented Pro residues and ALL in p8 were polar, aromatic with upwardly oriented Pro π bonds. There was also the striking rotamer finding that ALL VHLLAI and HIPI mHABPs p3χ1, p5χ2 and p7χ1 angles had *gauche* ^+^ orientation.

The foregoing structural, functional and immunological analysis of a large number of mHABPs led to postulating a generalised principle for vaccine development. While peptide backbone N and O atoms’ stereo-electron and topochemical characteristics (associated with PPII_L_ conformation) determine their capacity for establishing H-bonds with MHCII amino acid side-chain atoms to anchor and stabilise IMPIPS to the PBR, side-chain atoms having *gauche* ^*+*^ orientation in p3χ1, p5χ2 and p7χ1 and interacting with the TCR are the topochemical and electron characteristics determining VHLLAI and sterile complete protective immunity induction.

We have previously shown the development of chemically-synthesised VHLLAI and HIPI mHABPs based on highly specific steric-electron and topochemical features, such as a 26.5 ±3.5 Å distance [19,20] between the farthest mHABP residues fitting into MHCII molecule PBR p1 to p9. Specific peptide bond Φ and Ψ angle plane rotation to conform or display PPII_L_ structures to ensure appropriate peptide backbone O and N atom orientation for suitable mHABP anchoring to MHCII molecules (the immune system’s **first lock**). Here we describe for the first time that there must be specific topochemical orientation and steric-electron residues pointing upwards or away from the PBR to theoretically contact the TCR, a coordinated *gauche* ^*+*^ orientation to χ1 in p3, χ2 in p5 and χ1 p7 regarding their side chains to allow appropriate MHCII-p-TCR fitting and complex formation (**second lock** of the immune system) to induce compete fully-**protective definitive** immune responses.

Our subatomic, stereo-electron and topochemical analysis based always on in depth, seminal work by distinguished immunology, structuralist and chemistry groups, and involving a large panel of IMPIPS components as well as a deep analysis of the MHCII-p-TCR complex formation, has led to developing the IMPIPS concept and methods for a logical and rational methodology for multi-epitope, multistage, very high, long-lasting non-interfering, chemically-synthesised, minimal subunit-based IMPIPS vaccines (malaria being one of them), for human health and welfare.

## Materials and Methods

### Ethics Statement

This study was approved by the Fundación Instituto de Inmunología's animal ethics committee. The capture of *Aotus* monkeys (International Union for Conservation of Nature and Natural Resources (IUCN) status: least concern), the pertinent maintenance, immunisation challenge and research procedures have been authorised by the official Colombian environmental authority in the Amazonian region (CORPOAMAZONIA, resolutions 0066/Sep/2006, 0028/May/2010, 0632/Jun/2010 and 0042/Jan/2011 and previous authorisations beginning in 1982).

The US Committee on the Care and Use of Laboratory Animals’ guidelines were followed for all animal handling procedures, in turn complying with Colombian regulations for biomedical research (resolution 8430/1993 and law 84/1989). Monkeys at the station were numbered, sexed, weighed, given a physical-clinical exam and kept temporally in individual cages, prior to all experimental procedures. They were kept in controlled conditions regarding temperature (25°-30° centigrade) and relative humidity (83%), similar to those present in their natural environment. The monkeys’ diet was based on a supply of fruit typical of the Amazon region (i.e. such primates’ natural diet), vegetables and a nutritional supplement including vitamins, minerals and proteins. Environmental enrichment included visual barriers to avoid social conflict, feeding devices, some branches and vegetation, perches and habitat. Any procedure requiring animal handling was practiced by trained veterinary personnel and animals were submitted to sedation and analgesia procedures to reduce stress when necessary. The monkeys were cared for by expert veterinarians and biologists and supervised weekly by CORPOAMAZONIA veterinarians.

All individuals were released back into the Amazon jungle after the experimental procedures and 30–40 days of quarantine and clinical evaluation in optimal health conditions, as approved by CORPOAMAZONIA and in the presence of its officials.

### Synthetic peptides

Peptides were synthesised by the multiple-solid-phase system using the tert-butoxycarbonyl (t-Boc) strategy. Synthetic peptide polymers were produced by adding Cys and Gly residues to the N- and C-termini, oxidised to obtain high molecular weight polymers for immunisation, and named according to our laboratory’s serial numbering system.

### CSP, TRAP, AMA, MSP2 and EBA-175 cloning, sequencing, expression and purification of their recombinant fragments

The *P*. *falciparum* 3D7 strain CSP—and TRAP-encoding sequences (plasmoDB accession: PF13_0201 and PFC0210c, respectively) were selected for primer design. The CSP-rI amplified region (forward: 5’-ATGCAGGAATACCAGTGCTA-3’ reverse: 5’-ATCAGGATTACCATCCG-3’) encoded amino acids 21–103, including CSP 4383 cHABP; the CSP-rII amplified region (forward: 5’-ATGCACAATATGCCAAATGAC -3’ and reverse: 5’- ATTAAACACACTGGAACATT—3’) encoded amino acids 283–379, including CSP 4388 cHABP. The TRAP rII amplified region (forward primer: 5’- ATGGCAGGATCAGATAATAAATA -3’ reverse: 5’- ATTCCACTCGTTTTCTTCAG -3’) encoded amino acid 504 to 574, including TRAP 3347 cHABP. The products were cloned in pEXP-5-CT/TOPO vector (Invitrogen).

AMA-1, MSP-2 and EBA-175-Nt encoding sequences (plasmoDB accession: PF11_0344, PF3D7_0206800 and PF3D7_0731500, respectively) were selected for primer design. The AMA-1 amplified region (forward primer: 5’- ATGTGGACGGAATATATGGCAAA—3’ and reverse 5’—AGCAGTAGTAGCAATGTATGAT 3’) encoded amino acids 110 to 430 (AMA-1 rI), including AMA-1 4313 cHABP. The MSP-2 amplified region (forward primer: 5’- ATGAAAAATGAAAGTAAATATAGC—3’ and reverse 5’—GTTATTTTGAGTTTCTTTAT 3’) encoded amino acids 21 to 150 (MSP-2-rI), including MSP-2 4044 cHABP while the EBA-175-rI amplified region (forward primer: 5’- ATGGATATAAAAGAGAATGAAAAAT—3’ and reverse 5’- ATGTCCATAATCTAAAAAACTA–3’) encoded amino acids 26 to 244, including EBA-175-Nt 1758 cHABP. The EBA-175-rII amplified region (forward primer: 5’- ATGAAAATGAAAGGAAATGATAC—3’ and reverse 5’-TTCCCTTTTCGTACAATTAT—3’) encoded amino acids 1146 to 1406, including EBA-175-Ct 1815 cHABP. The products were cloned in pEXP-5-CT/TOPO vector (Invitrogen).

All recombinant proteins were expressed in *E*. *coli* BL21-Al (Invitrogen), following the manufacturer’s recommendations, purified by affinity chromatography and the fractions were pooled and quantified using a Micro BCA protein assay kit (Thermo Scientific, Meridian, USA). Expected protein molecular weight bands were observed in Coomassie blue staining and Western blot (CSP-rI 10.0 kDa, CSP-rII 11.0 kDa, TRAP-rII 8.5 kDa, AMA-1 38 kDa, MSP-2-rI 14.5kDa, EBA-175-rI 26.1 kDa and EBA-175- rII 31.5 kDa). We were unable to express the SERA protein in different vector systems; immunogenicity studies with this protein were thus not performed.

### Animals and immunisation

*Aotus* monkeys captured in the Amazon jungle were kept at our Primate Station in Leticia (Colombia) maintained according to Colombian National Institute of Health guidelines (Law 84/1989) and monitored weekly by CORPOAMAZONIA officials (the Colombian state entity monitoring environmental protection). All monkey’s sera were pretested by IFA for antibody presence against *P*. *falciparum* shizonts at 1:20 dilution and *P*. *falciparum* Spz (purchased from Sanaria Inc. Bethedsda USA) at 1:40 dilution and those positive returned to the jungle without further manipulations.

Seven or eight randomly-assigned (IFA negative) monkeys per group were subcutaneously immunised with 125 μg polymerised mHABPs mixture (**Table 1**) in monkey trials A-01/12; B/12, C/12 on day 0; this was homogenised with Freund’s Complete Adjuvant (FCA) for the first dose and Freund’s Incomplete Adjuvant (FIA) for the second (day 20) and third (day 40) doses. Nineteen monkeys were involved in the last trial (mixture D/2013) with the same immunisation schedule, each receiving 100 μg of each peptide included in the mixture. Blood samples (2ml) were drawn for immunological studies on day 0 (P0) before the first immunisation and 10 days after the second (II10) or the third (III10) and blood was drawn 60 (III60), 80 (III80) and 180 (III180) days after the 3^rd^ dose in some trials to assess these antibodies’ duration.

### Challenge and parasitaemia assessment

Mrz-derived mHABP immunised *Aotus*, as well as control monkeys, were infected with 500,000 *P*. *falciparum* FVO-strain (100% infective) parasitised RBC via femoral vein for challenge 60 days after the last immunisation to test *P*. *falciparum* infection under the most stringent and extreme conditions. The aforementioned infective dose is equivalent to 15 infectious mosquito bites, as might occur in Africa during the high transmission season. Each monkey’s parasitaemia was measured daily, starting on day 5 after challenge. Fluorescence was used for reading parasites in terms of the percentage of parasitised RBC on the slide following very sensitive accridine orange staining. Protection was defined as being the **complete absence** of parasites in blood during the 15 days of the experiment. Non-protected monkeys developed patent parasitaemia from day 5 or 6, reaching ≥ 5% levels between days 8–10.

All the monkeys were treated with paediatric doses of quinine after challenge. They were kept in quarantine for a further 30 days after the end of the experiment and released back into the jungle following CorpoAmazonia’s institutional approval and our Institute’s ethical committee’s evaluation (>90% of the *Aotus* being returned in excellent conditions, close to their capture place).

### Immunological studies

Spz IFA titres, pattern, location and WB analysis are explained in the Results section.

### cHABP and mHABP structural determination

The 3D structure of ~200 *P*. *falciparum*-derived native cHABPs and their corresponding mHABPs has been determined to date by ^1^H-NMR two-dimensional experiments (in solution) such as COSY, TOCSY and NOESY, using systematic resonance assignments developed by Wüthrich [102]. cHABP and mHABP analysis has revealed a wide variety of secondary structure elements for these peptides, confirming that aminoacid replacement had induced very strong secondary structure modifications [19,20,35].

Data regarding cHABP and mHABP 3D structures obtained in solution by ^1^H-NMR has already been published [18–20,36]; the present comparative study involves Ф, ψ, χ1 and χ2 χ3 and χ4 angles for each conformer (**Fig 1** showing all mHABPs described herein). Insight II software (Accellrys, Inc., USA) was used for analysing the group of conformers obtained by ^1^H-NMR restriction; the one having the lowest energy was chosen (numbered according to our Institute’s serial listing followed by a dot and its corresponding conformer number) and its data exported in. wrl format for 3D representation in Autodesk 3ds Max software (Autodesk, Inc., USA).

### Inter-atom interaction with HLA-DRβ1* molecules

HLA-DRβ1*0422/0404-**25608.37** and HLA-DRβ1*0301/1301-**10022.43** complexes were obtained by simply superimposing ^1^H-NMR-obtained mHABP structures on PDB templates (1J8H [72], 1A6A [25]), according to their corresponding allele obtained by X-ray crystallography and modified in some cases according to genotyped *Aotus* monkey amino acid sequences [36]. Insight II (2000) biopolymer module software (Accelrys Software Inc., USA), run on an Indigo 2 station (Silicon Graphics), was used for superimposing backbones on the PDB template. H-bonds were determined in the resulting model without further refinement, using the same software.

## Supporting Information

S1 Fig Interaction between IMPIPS and their corresponding HLA-DRβ1’s molecules. Distance measurements are given in Angstroms (Å) for IMPIPS backbone atoms and their corresponding HLA-DRβ1* lateral chain atoms involved in H-bonds or vdW interactions in complexes established with Spz or Mrz mHABP conformers.
(TIF)
