# Supplementary figures and images for: IMPIPS: The Immune Protection-Inducing Protein Structure Concept in the Search for Steric-Electron and Topochemical Principles for Complete Fully-Protective Chemically Synthesised Vaccine Development
Source: PLoS One. 2015 Apr 16;10(4):e0123249. doi: 10.1371/journal.pone.0123249 (PMC4400017; doi:10.1371/journal.pone.0123249)

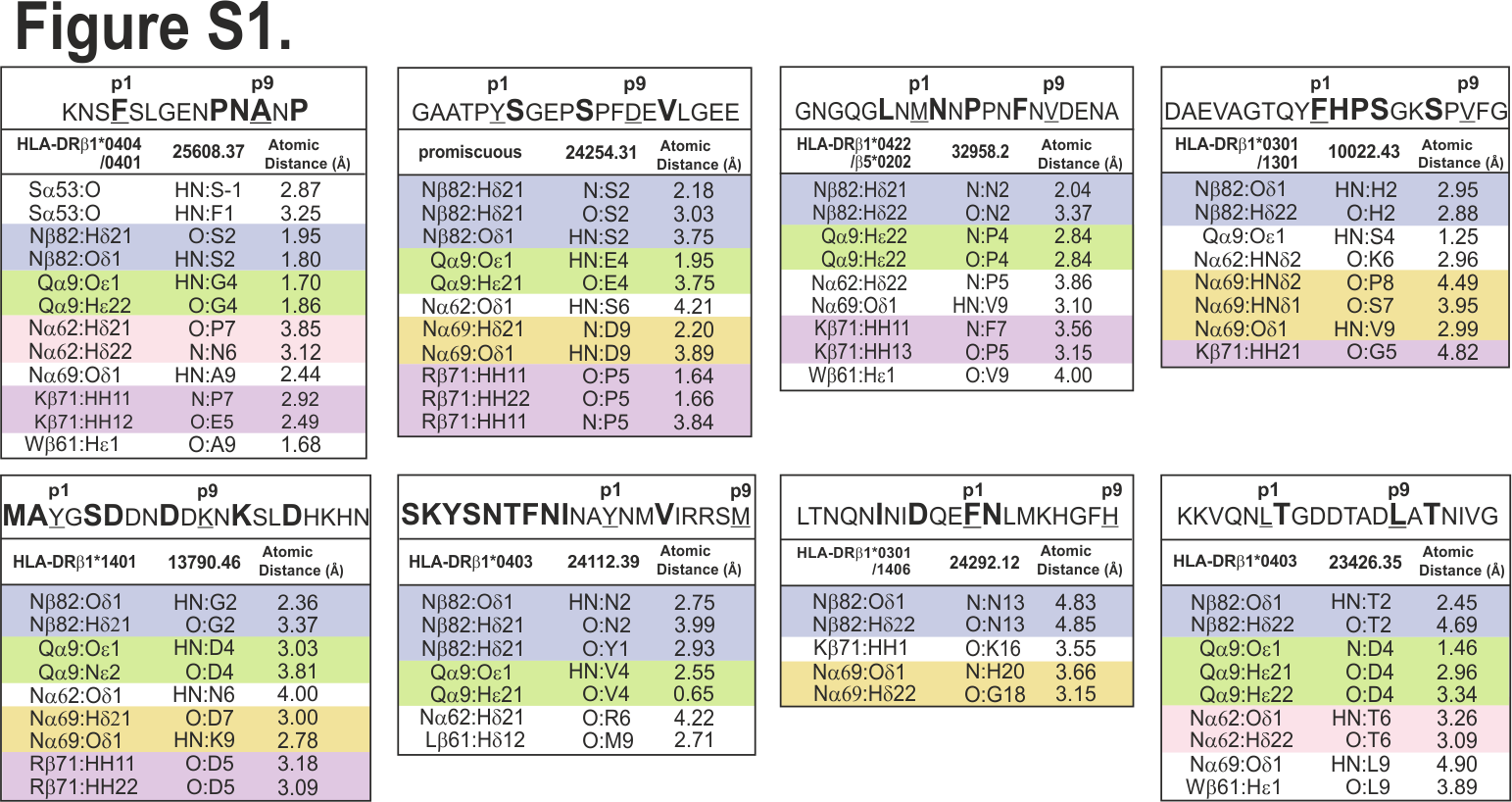

Supplement: S1 Fig — Distance measurements are given in Angstroms (Å) for IMPIPS backbone atoms and their corresponding HLA-DRβ1* lateral chain atoms involved in H-bonds or vdW interactions in complexes established with Spz or Mrz mHABP conformers. (TIF) [file pone.0123249.s001.tif]
